# Characterizing cellular receptor dynamics in live fluorescence imaging using stochastic modeling and machine learning

**DOI:** 10.1016/j.isci.2026.117435

**Published:** 2026-09-04

**Authors:** Giacomo Nardi, Matheus Santos Sano, Margaux Bilay, Anne Brelot, Jean-Christophe Olivo-Marin, Thibault Lagache

**Affiliations:** 1 Institut Pasteur, Université de Paris Cité, CNRS UMR 3691, BioImage Analysis Unit, 75015 Paris, France; 2 Institut Pasteur, Université de Paris Cité, CNRS UMR 3691, Dynamics of Host-Pathogen Interactions Unit, 75015 Paris, France

**Keywords:** cell receptors, TIRF imaging, stochastic motions, subdiffusion, Brownian motion, geometrical features, machine learning, random forest, CCR5, HIV

## Abstract

Cell surface receptor dynamics regulate signaling and endocytosis and can be quantified by single-particle tracking using total internal reflection fluorescence microscopy. We developed an interpretable machine-learning framework that combines geometric features of trajectories with a random forest classifier to distinguish five motion classes: Brownian motion, directed motion, fractional Brownian motion, Ornstein-Uhlenbeck motion, and continuous-time random walk. Applied to trajectories of the HIV co-receptor C-C chemokine receptor type 5 (CCR5), the method shows that stimulation with the agonist PSC-RANTES shifts receptor dynamics from intermittent and correlated diffusion toward confined, attraction-driven motion, consistent with receptor clustering and internalization. By providing accurate classification together with interpretable descriptors of receptor behavior, this framework links stochastic motion models to biologically meaningful cellular states. The approach enables quantitative analysis of complex receptor dynamics and is broadly applicable to live-cell imaging studies of membrane proteins and other single-particle tracking datasets.

## Highlights

• Explainable geometric features classify stochastic receptor trajectories
• Five biophysically relevant receptor motion states are distinguished
• PSC-RANTES shifts CCR5 from intermittent to confined membrane dynamics
• Motion signatures provide quantitative readouts of receptor functional states

## Graphical abstract

**Figure:**
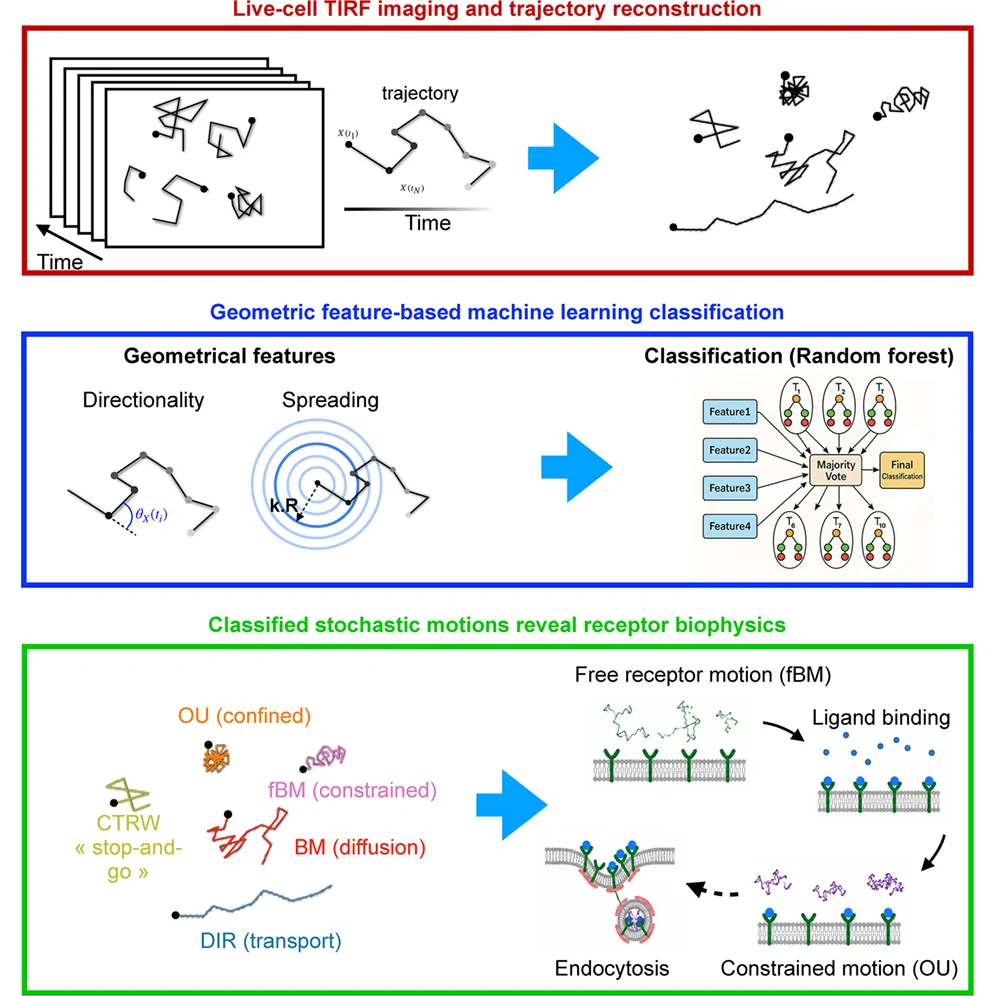

## Teaser

Biocomputational method; Cell biology; Computational bioinformatics

## Introduction

Cell receptors are essential in mediating the interaction between cells and external signals, such as the activation of inflammatory signaling pathways triggered by chemokine receptor binding^1^ or the internalization of molecules, including pathogens, into the cytoplasm. Viruses, for example, exploit this internalization process to gain access to the host cell’s replication machinery and proliferate.^2^ To gain a deeper understanding of receptor diversity and function *in vivo* and to develop therapeutics targeting the early stages of infection, a standard approach involves labeling specific receptors with fluorescent probes followed by live time-lapse fluorescence imaging. Total internal reflection fluorescence microscopy (TIRFM) has emerged as an effective imaging technique, as its limited imaging depth—restricted to the first ∼200 nm of the plasma membrane—helps minimize background noise from the cell interior.^3^

Receptor dynamics provide valuable insights into the biophysical constraints influencing receptor behavior, such as confinement within membrane microdomains, receptor clustering, and chemical interactions with ligands.^4^ Therefore, it is crucial to automatically track individual fluorescently labeled receptors and accurately characterize their dynamic behavior.

Although several methods^5^^,^^6^ enable the trajectory reconstruction of labeled molecules in time-lapse fluorescence imaging, motion classification still lacks robust approaches for capturing the complexity of stochastic dynamics. Motion classification is typically developed within a diffusion-based framework that enables the detection of three primary types of motion^4^^,^^7^^,^^8^: *Brownian* motion (BM), which describes free diffusion of molecules^9^; *subdiffusive* motion, which characterizes constrained evolution due to obstacles, crowding, or chemical potentials^10^^,^^11^; and *superdiffusive* motion, which accounts for active transport driven by molecular motors.^12^^,^^13^^,^^14^

However, this diffusion-based approach cannot differentiate between distinct co-existing subdiffusive behaviors^15^ that govern receptor dynamics and their interactions with ligands. Receptor dynamics are influenced by the viscoelastic properties of the cellular medium, which result in constrained and crowded movement.^16^ Additionally, specific patterns emerge, such as intermittent motions due to molecular trapping or confined paths associated with internalization.^17^^,^^18^ Consequently, distinguishing these stochastic behaviors^19^^,^^20^^,^^21^^,^^22^ presents a significant challenge for motion classification algorithms.

Beyond diffusion-based categories that rely primarily on anomalous diffusion exponents, recent efforts have shifted toward *process-based classification*, in which individual trajectories are explicitly assigned to underlying stochastic motion models, thereby capturing mechanistic differences that cannot be resolved from diffusion scaling alone.^8^^,^^19^^,^^23^ This paradigm aims to identify the physical mechanisms underlying the observed dynamics, which is essential for interpreting receptor behavior in a biological context.

The second challenge concerns the definition of robust descriptors discriminating the different types of stochastic motions. The diffusion-based classification traditionally uses the mean squared displacement (MSD) function and discriminates the diffusion class based on its polynomial order.^24^^,^^25^ However, the MSD criterion has many drawbacks, linked to the accurate statistical estimate of the fitting coefficient (see supplemental information), which has encouraged the introduction of other analytical descriptors^26^^,^^27^ such as the Gaussianity of displacement distribution,^28^ statistics on the displacement traveled by a particle,^4^^,^^7^^,^^29^^,^^30^ the moment spectrum analysis,^31^^,^^32^ or the trajectory *p*-variation.^19^

Classification algorithms build on these features to define statistical hypothesis tests^4^^,^^7^^,^^19^^,^^29^ or machine learning (ML) approaches.^23^^,^^33^ Most feature-based ML approaches remain embedded in a diffusion-based labeling scheme and therefore do not explicitly discriminate between distinct stochastic processes that can share similar diffusive properties.

To avoid the need for defining *ad hoc* features, recent deep learning approaches have been developed,^21^^,^^34^^,^^35^^,^^36^ primarily within the context of the anomalous diffusion (AnDi) challenge,^22^ which aims to benchmark algorithms for classifying anomalous diffusion models. While these methods achieve high classification accuracy, they are predominantly black-box models and often focus on non-ergodic dynamics, limiting both interpretability and applicability to receptor dynamics at the cell membrane, where directed and confined motions are essential.

In this work, we first generate trajectories from five canonical stochastic processes commonly used to model receptor dynamics. We then compute four interpretable geometric features from each trajectory and evaluate their ability to discriminate between motion types using a random forest classifier. Finally, we compare the proposed framework with existing trajectory-classification approaches and apply it to experimental single-particle tracking data.

A key strength of our approach is its ability to jointly discriminate between five distinct stochastic processes governing receptor dynamics at the cell membrane: BM, directed motion (DIR), and three subdiffusive processes, namely fractional Brownian motion (FBM),^37^ Ornstein-Uhlenbeck (OU) dynamics,^38^ and continuous-time random walks (CTRWs).^39^ These processes were chosen because they capture distinct and complementary biophysical mechanisms relevant to receptor dynamics, including confinement (OU), viscoelasticity (FBM), and intermittent trapping (CTRW).

Previous studies have typically addressed subsets of these stochastic processes in isolation and within heterogeneous methodological frameworks. In particular, benchmark efforts such as the AnDi challenge^22^ focus primarily on FBM and CTRW and do not explicitly consider DIR or OU dynamics. Although several analytical descriptors have been proposed to characterize individual processes,^8^^,^^19^^,^^23^ no existing approach can discriminate such a broad family of stochastic motions within a single, interpretable framework. By combining process-based classification with explainable geometric features, our method advances beyond both diffusion-based feature approaches and deep-learning models developed in the AnDi context, while remaining directly applicable to experimental receptor tracking data.

After thoroughly validating our method with synthetic data, we applied it to analyze a set of C-C chemokine receptor type 5 (CCR5) trajectories at the cell membrane, obtained through TIRFM . CCR5 plays a critical role in inflammatory responses and human immunodeficiency virus (HIV) infection, serving as a co-receptor for the virus to enter and infect host cells.

Using our feature-based ML approach, we uncovered a complex landscape of subdiffusive dynamics in the CCR5 trajectories. In the basal state, the majority of tracks exhibited either intermittent motion (CTRW) or continuous dynamics within a constrained environment (FBM). However, upon stimulation with PSC-RANTES, an analog of the natural ligand RANTES/CCL5 with potent anti-HIV-1 activity, we observed a marked redistribution of motion classes, with basal dynamics dominated by CTRW and FBM giving way to more confined, attraction-driven OU dynamics. Our method thus reveals that PSC-RANTES stimulation shifts receptor behavior from a heterogeneous regime characterized by intermittent and correlated motion toward constrained dynamics at the cell membrane, consistent with receptor clustering and subsequent internalization. This highlights the critical importance of distinguishing a broader range of stochastic dynamics, particularly in the subdiffusive regime, to better understand the relationship between receptor movement and activation in the context of cell signaling.

## Results

**Figure 1:**
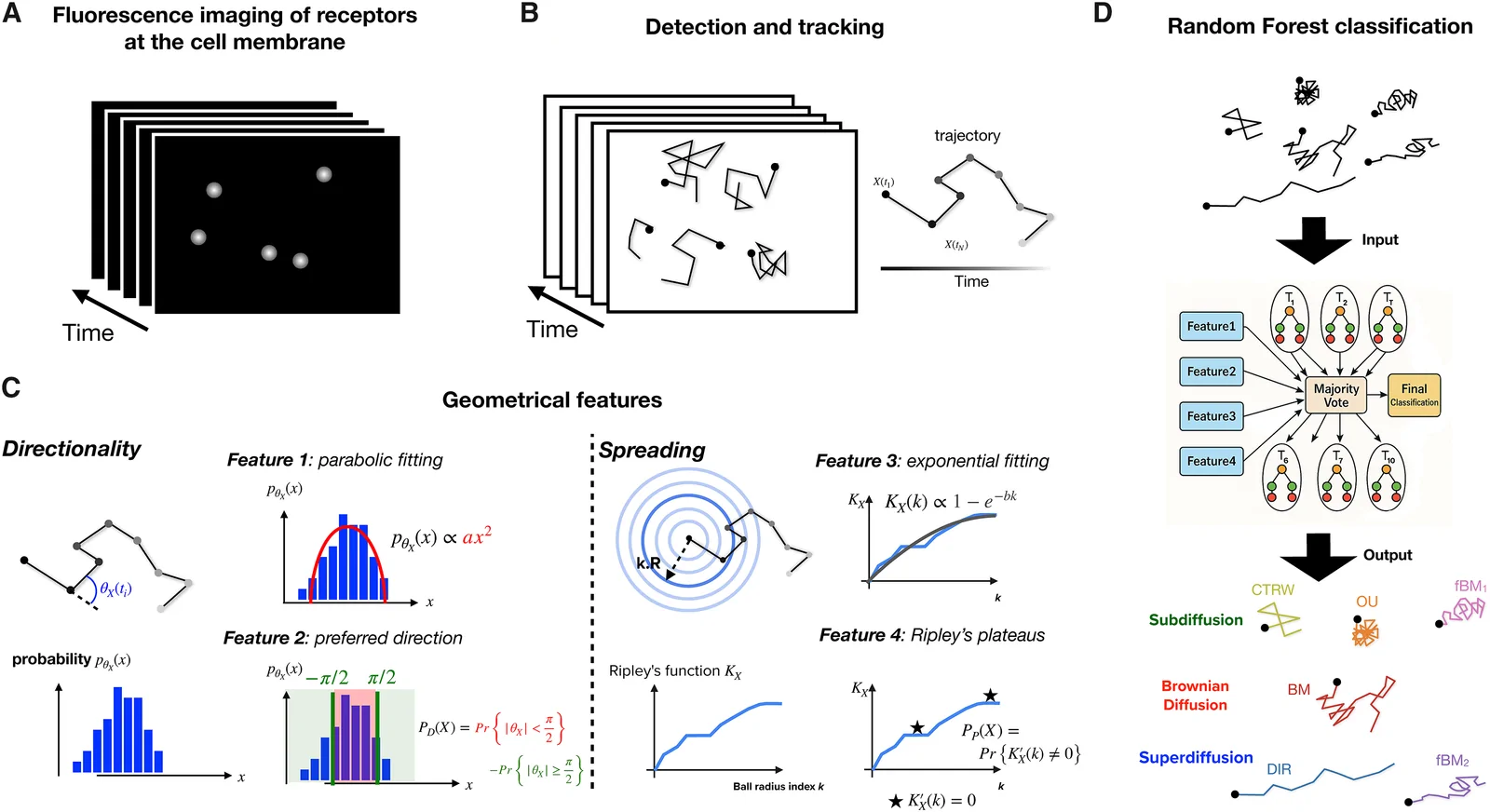
Overview of the trajectory classification pipeline (A) Time-lapse fluorescence imaging (e.g., TIRF microscopy) of membrane receptors at the cell surface. (B) Automatic detection of individual fluorescent spots and reconstruction of single-particle trajectories by single-particle tracking across successive frames. (C) Extraction of four geometrical features from each trajectory *X*: two features quantify *directionality* and two quantify *spatial spreading*. Feature 1 (curvature *a*) measures the curvature of the histogram of successive turning angles {*θ*_*X*_(*t*_*i*_)} via a parabolic fit *ax*^2^. Feature 2 (preferred direction *P*_*d*_) quantifies directional persistence as $P_{d}\left(X\right)=P\left(|\theta_{X}|<\pi /2\right)-P\left(|\theta_{X}|\geq \pi /2\right)$. Feature 3 (rate *b*) characterizes spatial spreading through an exponential fit 1 − e^−*bk*^ of Ripley’s vector components *K*_*X*_(*kR*) (Equation 8). Feature 4 (plateau proportion *P*_*p*_) measures the fraction of scales *k* for which the discrete derivative of *K*_*X*_(*kR*) vanishes, capturing spatial confinement. (D) The four features are used as inputs to a random forest classifier (10 decision trees), which assigns each trajectory to one of five stochastic motion classes: BM, DIR, OU, FBM, or CTRW. In our simulations, CTRW and OU correspond to subdiffusive dynamics, DIR to superdiffusive dynamics, and FBM may be sub- or superdiffusive depending on the Hurst exponent.

We develop a feature-based machine-learning approach for classifying stochastic receptor motion at the cell membrane (Figure 1). We begin by evaluating the performance of our method on simulated trajectories (Table 1; see STAR Methods section). Specifically, we examine the impact of localization error and variability in trajectory length on classification accuracy. Next, we compare our approach to three state-of-the-art methods: a statistical classification technique,^7^ a method based on moment scaling spectrum (MSS) analysis of particle displacements,^31^ and a deep learning model trained on raw trajectories.^40^

**Table 1:** Parameter ranges used to simulate the five stochastic motion classes for the training and evaluation datasets

Simulated process	Simulation parameters
Brownian motion (BM)	*σ* ∈ [0.1, 10]
Ornstein-Uhlenbeck (OU)	$\sigma \in \left[1,\;10\right],\;\theta =\left(0,0\right),\;\lambda =\frac{r\sigma }{\sqrt{\Delta t}},\;r\in \left[0.2,\;1\right]$
Directed motion (DIR)	$\sigma \in \left[1,\;10\right],\;\mu =\frac{r\sigma }{\sqrt{2\Delta t}}\;\left(1,1\right),\;r\in \left[0.2,\;1\right]$
Fractional Brownian motion (FBM)	*H* ∈ [0.15, 0.30] ∪ [0.70, 0.85]
Continuous-time random walk (CTRW)	*σ* ∈ [0.1, 10], *γ* ∈ (0, 1)

The parameters were sampled uniformly over the indicated intervals.

After establishing its accuracy and resilience through extensive simulations, we apply the method to experimental biological data to investigate the dynamic behavior of CCR5 receptors at the cell membrane.

### Classification of simulated trajectories

#### Method accuracy increases with trajectory length

Tables 2 and 3 summarize the method’s accuracy as a function of trajectory length *N*. Since the sampling interval (Δ*t*) is fixed, varying *N* effectively changes the duration of the observed trajectory while preserving the temporal resolution. The analysis presented in Tables 2 and 3 therefore evaluates how the amount of trajectory information available for classification influences performance. As expected, accuracy improves with longer trajectories, exceeding 90% for lengths *N* > 70. This reflects the fact that directional and spreading characteristics require time to emerge clearly in particle motion.

**Table 2:** Accuracy $\left(\frac{True\;Positive+True\;Negative}{Positive+Negative}\right)$ and recall $\left(\frac{True\;Positive}{True\;Positive+False\;Negative}\right)$ for datasets composed of 5,000 simulated trajectories of length *N*

Length	Accuracy	BM	OU	DIR	FBM	CTRW
*N* = 40	80.3 ± 1.1	80.1 ± 1.1	78.5 ± 1.1	81.5 ± 1.1	61.6 ± 1.3	99.9 ± 0.1
*N* = 70	90.0 ± 0.8	87.3 ± 0.9	88.3 ± 0.9	89.7 ± 0.8	79.6 ± 1.1	100.0 ± 0.0
*N* = 100	93.3 ± 0.7	93.2 ± 0.7	91.2 ± 0.8	92.8 ± 0.7	89.3 ± 0.9	100.0 ± 0.0
*N* = 200	98.1 ± 0.4	98.4 ± 0.3	97.7 ± 0.4	98.0 ± 0.4	96.3 ± 0.5	99.9 ± 0.1
*N* = 300	99.2 ± 0.2	98.8 ± 0.3	99.1 ± 0.3	99.3 ± 0.2	98.9 ± 0.3	100.0 ± 0.0

The table reports the overall classification accuracy together with the recall for each motion class. Values are reported as mean ± 95% binomial confidence interval (M = 10 independent simulations for each trajectory length).

**Table 3:** Precision $\left(\frac{True\;Positive}{True\;Positive+False\;Positive}\right)$ by motion class for different trajectory lengths *N*

Length	BM	OU	DIR	FBM	CTRW
*N* = 40	69.3 ± 1.3	79.9 ± 1.1	81.3 ± 1.1	71.5 ± 1.3	100.0 ± 0.0
*N* = 70	81.5 ± 1.1	89.0 ± 0.9	87.5 ± 0.9	87.3 ± 0.9	100.0 ± 0.0
*N* = 100	88.6 ± 0.9	93.1 ± 0.7	94.0 ± 0.7	91.0 ± 0.8	100.0 ± 0.0
*N* = 200	95.8 ± 0.6	97.8 ± 0.4	98.0 ± 0.4	98.8 ± 0.3	100.0 ± 0.0
*N* = 300	98.9 ± 0.3	99.2 ± 0.2	98.8 ± 0.3	99.2 ± 0.2	100.0 ± 0.0

Errors represent the corresponding binomial proportion confidence intervals (95% confidence level; M = 10 simulations per trajectory length).

Tables 2 and 3 report the recall and precision of the method for each type of stochastic process and trajectory length.

Most misclassifications occur for short FBM trajectories, which are often confused with OU or BM for *N* = 40 and *N* = 70. This is likely due to the broad spectrum of dynamical behaviors exhibited by FBM, which depends on the Hurst exponent *H*. FBM is intrinsically harder to classify for short or noisy trajectories, as identifying its long-range temporal correlations requires sufficiently long and reliable trajectories. However, this confusion decreases markedly with longer trajectories, confirming that FBM captures fundamentally distinct dynamics when sufficient temporal information is available.

**Figure 2:**
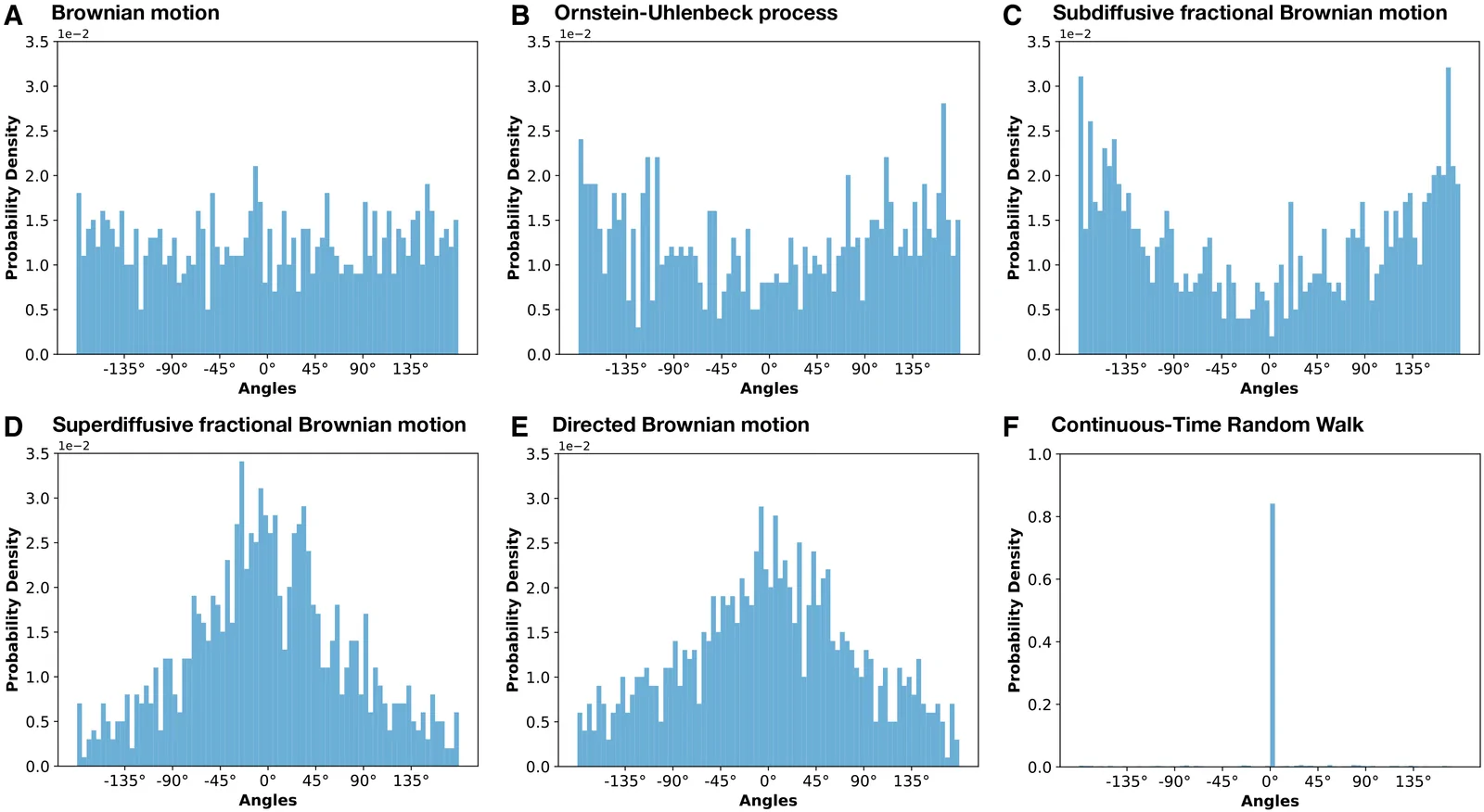
Angle distribution for different processes (A–F) Examples of angle distribution (probability density function, Equation 3) are shown for different simulated processes (*N* = 1,000, Δ*t* = 1) (A) BM (*σ* = 1). (B) OU (*λ* = 0.5, *σ* = 1). (C) FBM (*H* = 0.2). (D) FBM (*H* = 0.8). (E) DIR (‖*u*‖ = 0.7, *σ* = 1). (F) CTRW (*σ* = 1 and *γ* = 0.9).

In contrast, CTRW is reliably identified even for short trajectories. This robustness stems from the characteristic waiting times and the distinctive features extracted from the angle histogram $p_{\theta_{X}}$ (Equation 3 Figure 2) and the Ripley function *K*_**X**_ (Equation 8).

**Figure 3:**
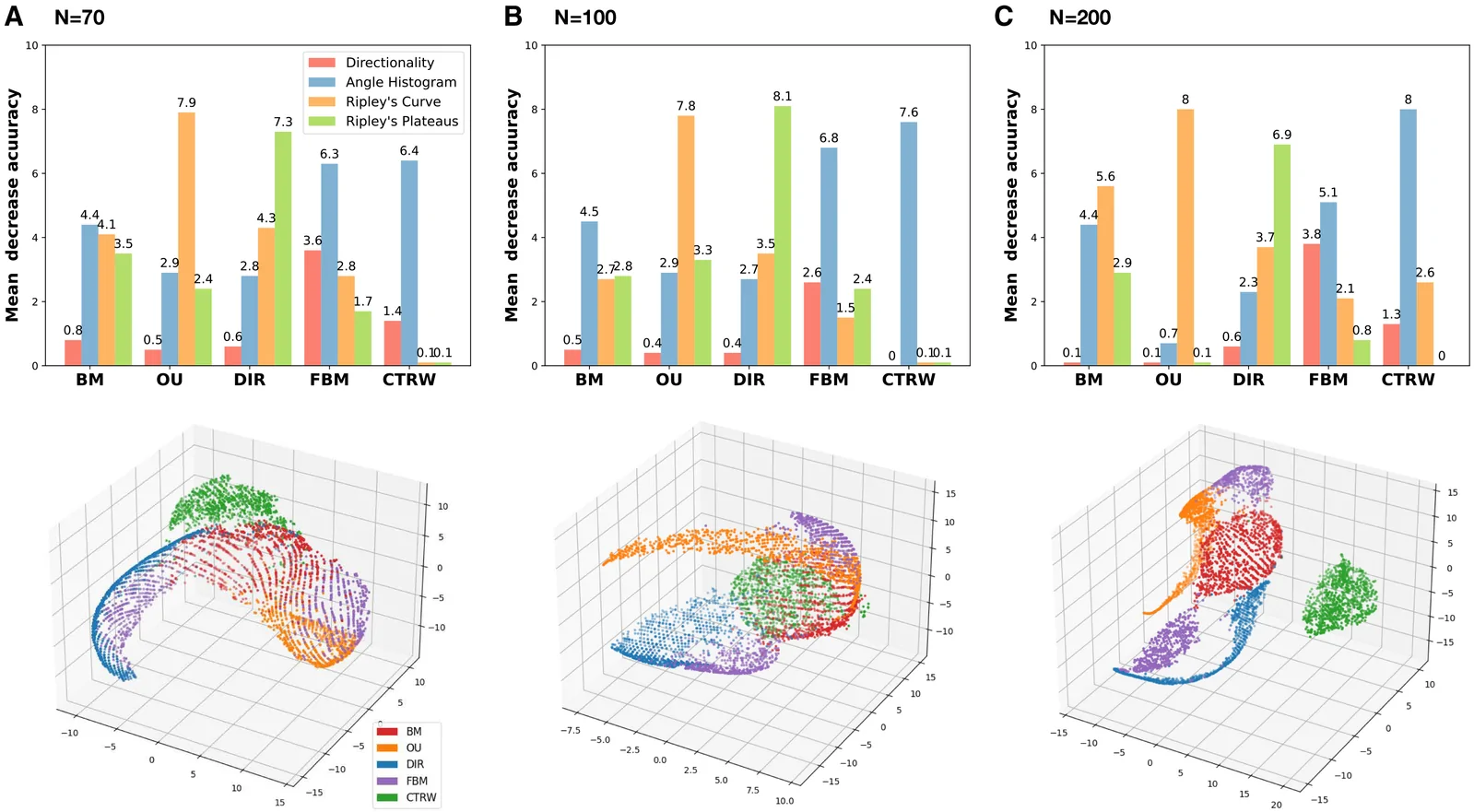
Feature importance and class separability for different trajectory lengths (A–C) Top: mean decrease in recall (in %) for each motion class (BM, OU, DIR, FBM, and CTRW) induced by random permutation of individual feature values, computed from a random forest classifier trained on simulated trajectories. The four features correspond to directionality, angle histogram, Ripley’s *K*-function curve, and Ripley’s *K*-function plateau. Bars represent the average recall drop over 10 independent permutations, providing a measure of feature importance for classification. Results are shown for three trajectory lengths: (A) *N* = 70, (B) *N* = 100, and (C) *N* = 200 steps. Bottom: three-dimensional t-SNE embedding of the four-dimensional feature space (directionality, angle histogram, Ripley’s curve, and Ripley’s plateau) for the same datasets, colored by motion class. Each point corresponds to one trajectory, and the projections illustrate the degree of separability between motion classes for the different trajectory lengths.

We next analyze the relative importance of each geometrical feature (Table 4) for trajectory classification as a function of motion type. To visualize how these features distinguish between stochastic motion classes, we first apply a 3D t-distributed stochastic neighbor embedding (t-SNE)^41^ to the extracted features across simulated motion types (Figure 3). The t-SNE algorithm reduces high-dimensional data into a low-dimensional embedding that preserves local density relationships. The resulting projections show clear separation between motion classes for different trajectory lengths (*N* = 70, 100, and 200), indicating that the proposed geometrical features can robustly discriminate between the modeled dynamics.

**Table 4:** Summary of the four geometrical features considered in this work together with their corresponding geometrical meaning and biological interpretation

Feature	Geometrical meaning	Biological interpretation
Parabolic fitting (Equation 4)	motion amplitude	trajectory oscillations
Preferred direction (Equation 5)	preferred direction	forward/backward motions
Exponential fitting (Equation 9)	explored region	motion confinement
Ripley’s plateaus (Equation 10)	local confinement	particle trapping

To assess the contribution of each feature to the classification task, we perform a permutation-based mean decrease in accuracy (MDA) analysis. For each feature, we randomly permute its values across the dataset and evaluate the change in recall for each motion class using the trained random forest model. We used a random forest classifier as it provides a good trade-off between interpretability and the ability to model non-linear relationships in the data, leading to robust performance with minimal hyperparameter tuning. This procedure is repeated ten times to average out stochastic effects. The recall differences (relative to the unperturbed dataset) are displayed as bar plots in the top row of Figure 3, showing the per-class importance of each feature.

**Figure 4:**
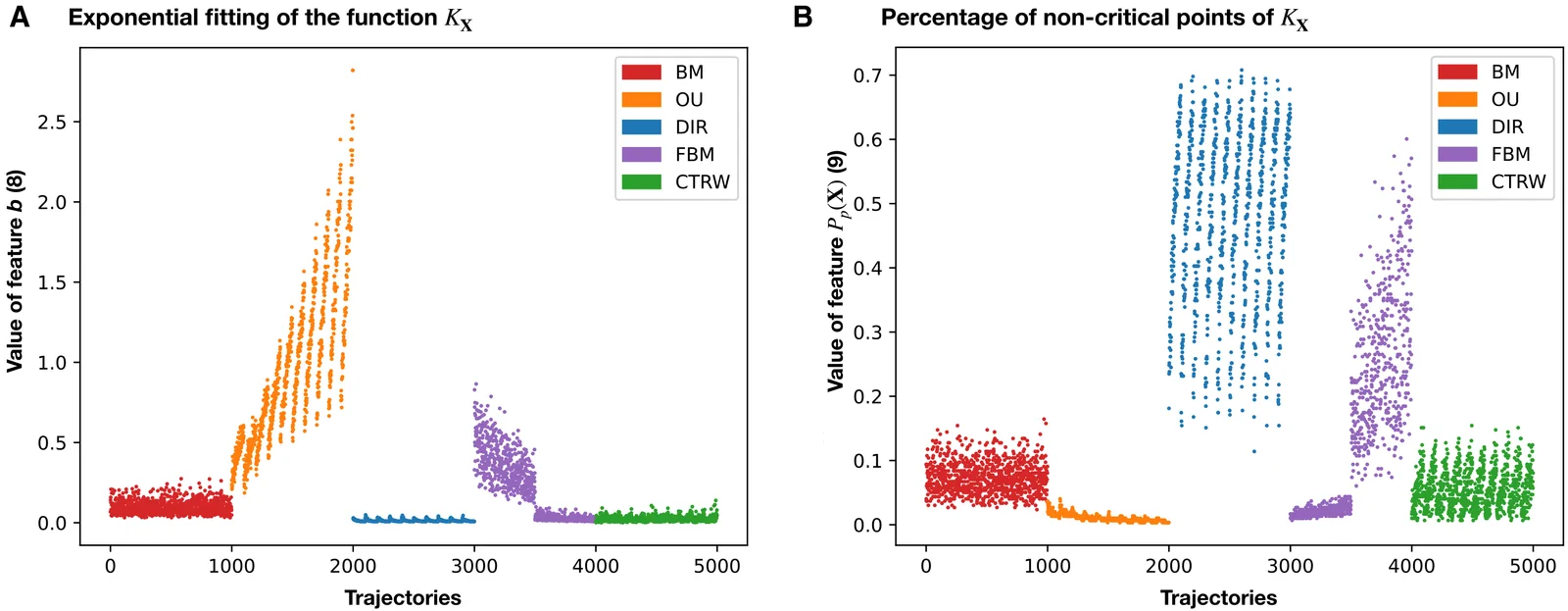
Features encoding trajectory spreading Parameters describing the analytical form of Ripley’s curve *K*_*X*_ are shown for simulated trajectories from the different dynamics (*N* = 300, Δ*t* = 1/30 *s*, 1,000 trajectories per motion type). Each point corresponds to one trajectory; colors indicate the underlying stochastic model: BM, OU, DIR, FBM, and CTRW. (A) Distribution of the exponential fitting parameter *b* (Equation 9). (B) Distribution of the percentage *P*_*p*_(*X*) of *K*_*X*_ points with a non-zero derivative (Equation 10). Two regimes of fractional Brownian motion (FBM) are represented: a subdiffusive regime (*H* < 1/2) and a superdiffusive regime (*H* > 1/2). Both are considered as FBM trajectories in the classification framework.

Although feature importance varies with trajectory length, several consistent trends emerge. The Ripley curve fitting coefficient *b* (Equation 9 Figure 4) plays a key role in identifying OU processes. This is due to the confined nature of OU trajectories, which results in a rapid saturation of the Ripley curve (see STAR Methods). The restoring force acting on the particle (or receptor) confines its motion within a potential well, preventing wide spatial exploration. As a result, the initial Ripley vector (Equation 8) rapidly approaches its maximum value of 1.

**Figure 5:**
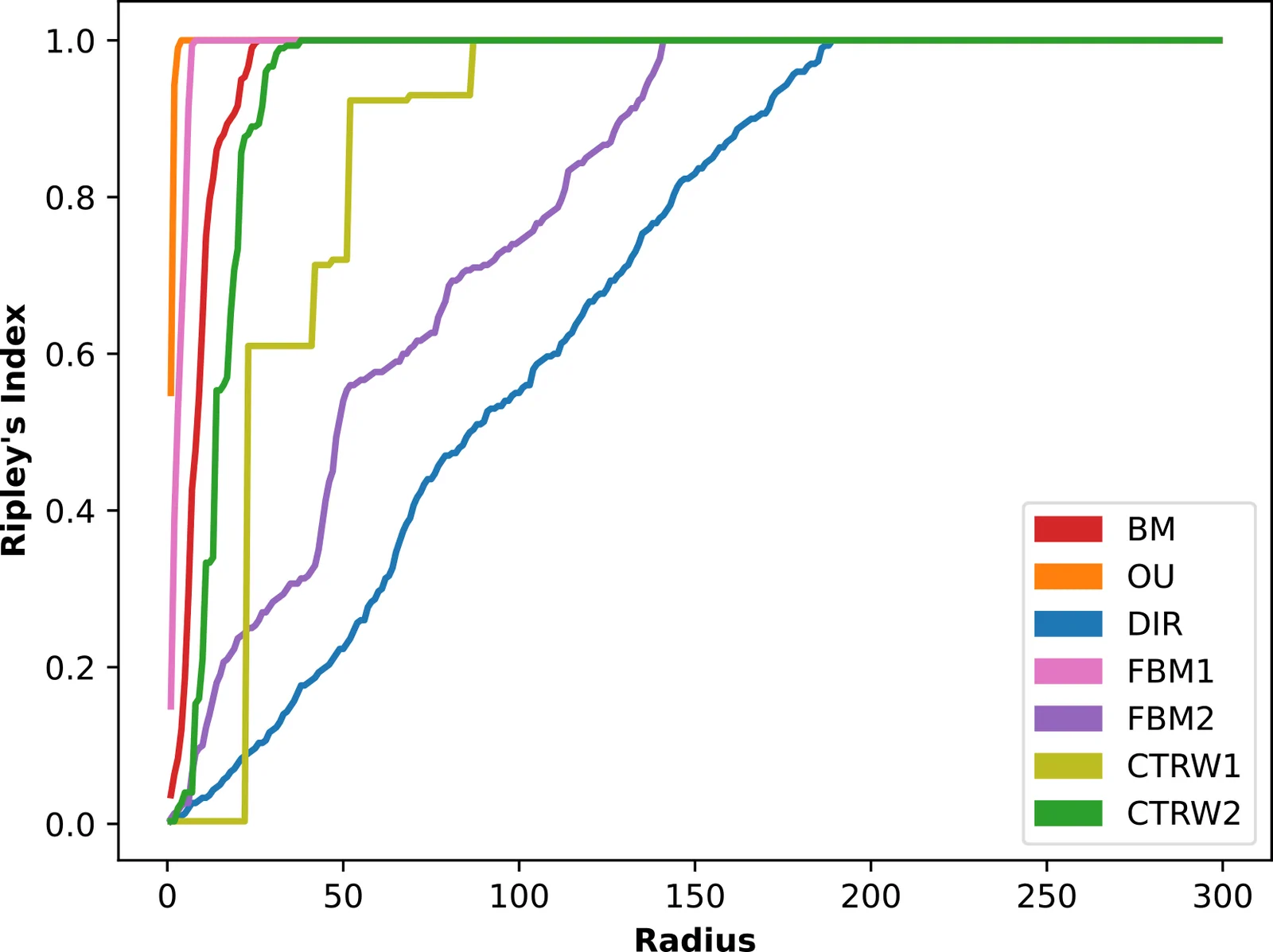
Ripley’s index for different simulated processes The Ripley’s index (Equation 6) is plotted for different simulated stochastic processes (*N* = 300, Δ*t* = 1). The radius in the *x* axis is expressed in terms of index 1 ≤ *k* ≤ *N* (Equation 8). Parameters for simulated processes are: BM (*σ* = 1), OU (*λ* = 0.5, *σ* = 1), DIR (‖*u*‖ = 0.7, *σ* = 1), FBM (FBM1 with *H* = 0.2 and FBM2 with *H* = 0.8), and CTRW (*σ* = 1, *γ* = 0.01 for CTRW1 and *γ* = 0.9 for CTRW2).

In contrast, the Ripley plateau measure *P*_*p*_(**X**) (Equation 10) is particularly effective in identifying DIR motion. Intuitively, plateaus in the Ripley *K*-function reflect spatial confinement, and *P*_*p*_(**X**) quantifies this effect by measuring the proportion of points where the derivative of *K* vanishes: larger values indicate stronger confinement, whereas smaller values correspond to more freely spreading trajectories. This behavior is illustrated in STAR Methods Figure 5, where confined motions exhibit extended plateaus in the Ripley curve, in contrast to spreading motions for which the curve remains strictly increasing. In comparison, DIR trajectories, driven by a persistent drift component, display a monotonically increasing Ripley index with few or no plateaus.

CTRW trajectories are primarily characterized by their angular histogram $p_{\theta_{X}}$ (Equation 3), which often resembles a sharp peak (approaching a Dirac delta) centered at zero due to frequent pauses and reorientations. In contrast, BM and FBM display more uniform distributions across features. Notably, FBM classification is most sensitive to the directionality feature, which effectively differentiates it from both OU (confined) and DIR (drift-dominated) processes.

These results highlight the dual strength of our approach. First, the selected features encode intrinsic geometrical properties of each stochastic process, enabling interpretable, feature-based classification. Second, this geometrical representation supports a more nuanced view of motion, extending beyond the classical diffusion paradigm and allowing direct connections to biological mechanisms and constraints.

#### Impact of localization error

In fluorescence microscopy, localization errors can significantly affect the accurate positioning of tracked particles. Estimating these errors is challenging, as they depend on the optical setup^42^ and on specific live-cell imaging conditions, such as thermal and mechanical fluctuations of the cell membrane.^43^ Nonetheless, quantifying localization error is crucial for trajectory analysis—especially in the case of CTRW, where accurate detection of particle immobility is essential.

To evaluate the robustness of our method to localization errors, we model the noise as a zero-mean Gaussian process with variance *σ*^2^. We generate perturbed datasets by adding this noise to each position in the simulated trajectories. Following the approaches of Wagner et al.^23^ and Janczura et al.,^33^ we define a *localization confidence* *L*_*c*_ as the ratio between the particle’s expected displacement (from diffusion and drift) and the standard deviation of the localization noise:

(Equation 1) $$L_{c}=\left\{\begin{array}{cc} \frac{\sqrt{D\Delta t+\mu^{2}{\left(\Delta t\right)}^{2}}}{\sigma } & \text{for}\;\text{DIR}\\ \frac{\sqrt{D\Delta t}}{\sigma } & \text{otherwise} \end{array}\right)$$

Here, *D* is the diffusion coefficient, estimated by fitting the MSD to the form MSD(*t*) ∝ 4*Dt*^*α*^, and *μ* is the magnitude of the drift component for DIR.

By varying *L*_*c*_ from 1 (low confidence, i.e., high localization error) to 9 (high confidence, i.e., low localization error), we determine the corresponding noise amplitudes *σ*(*L*_*c*_) and create datasets with increasing levels of localization noise. Simulations were performed at the micrometer scale using diffusion coefficients in units of *μ*m^2^/s and a frame interval of $\Delta t=\frac{1}{30}\;s\approx 33.3\;ms$.

Experimentally, receptor diffusion coefficients typically lie in the range *D* ≈ 0.1–1.0 *μ*m^2^/s.^44^^,^^45^^,^^46^ Defining the localization error for confidence level *L*_*c*_ ∈ {1, *…*, 9} as

$$\sigma =\frac{\sqrt{D\;\Delta t}}{L_{c}},$$

we obtain values ranging from *σ* = 6 nm (*D* = 0.1 *μ*m^2^/s, *L*_*c*_ = 9) to *σ* = 183 nm (*D* = 1.0 *μ*m^2^/s, *L*_*c*_ = 1). These estimates agree with localization errors reported for standard fluorescence microscopy techniques.^47^

To assess robustness, we train our classification model on clean (non-perturbed) trajectories of length *N* = 100 and test its performance on the noise-perturbed datasets at various *L*_*c*_ values.

Because localization noise can obscure true particle immobility—particularly relevant for CTRW classification—we implement a correction by defining an immobility threshold. At each time point, if the displacement from the previous position is below $\sqrt{2}\sigma \left(L_{c}\right)$, the point is reassigned to the previous location. This effectively filters out small displacements attributable to localization noise.

**Figure 6:**
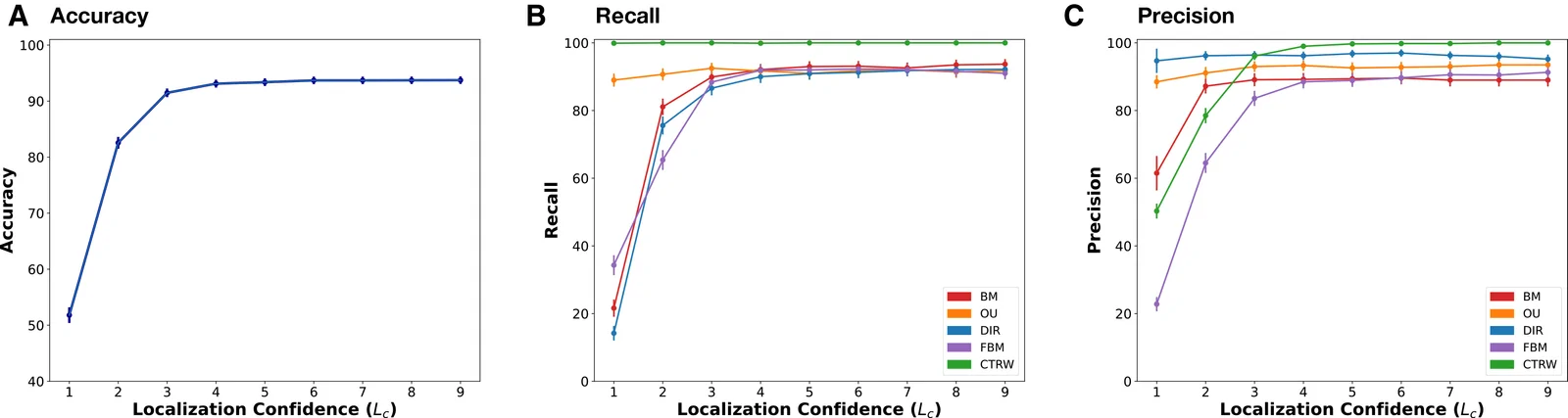
Classification robustness to localization uncertainty (A–C) (A) Overall accuracy, (B) recall, and (C) precision for each motion class (BM, OU, DIR, FBM, and CTRW). The method is trained on non-perturbed trajectories (*N* = 100, Δ*t* = 1/30) and tested on trajectories affected by localization error for different values of the localization confidence *L*_*c*_ (see Equation 1). The immobility correction is applied using a threshold of $\sqrt{2}\sigma \left(L_{c}\right)$. Error bars indicate 95% binomial proportion confidence intervals (*M* = 10 simulations per *L*_*c*_ value).

Figure 6 shows the classification performance on the perturbed datasets. Overall accuracy exceeds 90% for *L*_*c*_ ≥ 3, and both precision and recall remain above 80% across all motion types in this range. At low localization confidence (*L*_*c*_ = 1, 2), CTRW and OU trajectories are still classified accurately, but performance deteriorates for other motion types. BM and DIR show low recall but high precision, indicating that while few false positives occur, many true instances are missed. In contrast, FBM exhibits both low recall and low precision, suggesting frequent misclassifications and poor detection.

The reduced precision for CTRW at low *L*_*c*_ reflects an overestimation of immobility—particularly for BM and FBM trajectories. This is consistent with the fact that, at low *L*_*c*_, the diffusion coefficient used in simulation approaches the immobility threshold $\sqrt{2}\sigma \left(L_{c}\right)$. As *L*_*c*_ increases, the diffusion coefficient becomes significantly larger than the noise variance *σ*^2^, mitigating immobility overestimation and improving classification accuracy.

In conclusion, our analysis shows that for *L*_*c*_ ≥ 3, classification performance on noisy trajectories closely matches the accuracy reported in Tables 2 and 3 for clean trajectories of length *N* = 100. This confirms that the classification algorithm is robust to the levels of localization error typically encountered in fluorescence microscopy, ensuring its reliability for experimental applications.

#### Impact of trajectory length mismatch between training and testing

The proposed method is designed to classify trajectories of a fixed length *N*, which introduces a practical challenge when applying it to experimental data: obtaining trajectories of uniform length requires significant effort in sorting the acquired paths and often demands a larger number of acquisitions. This is particularly problematic in real experimental settings, where particles may appear at different life stages and follow independent fates—some exiting the field of view, others entering it. Additionally, factors such as particle density and temporal fluorescence variability may prevent consistent detection over time, resulting in a wide range of trajectory lengths.

**Figure 7:**
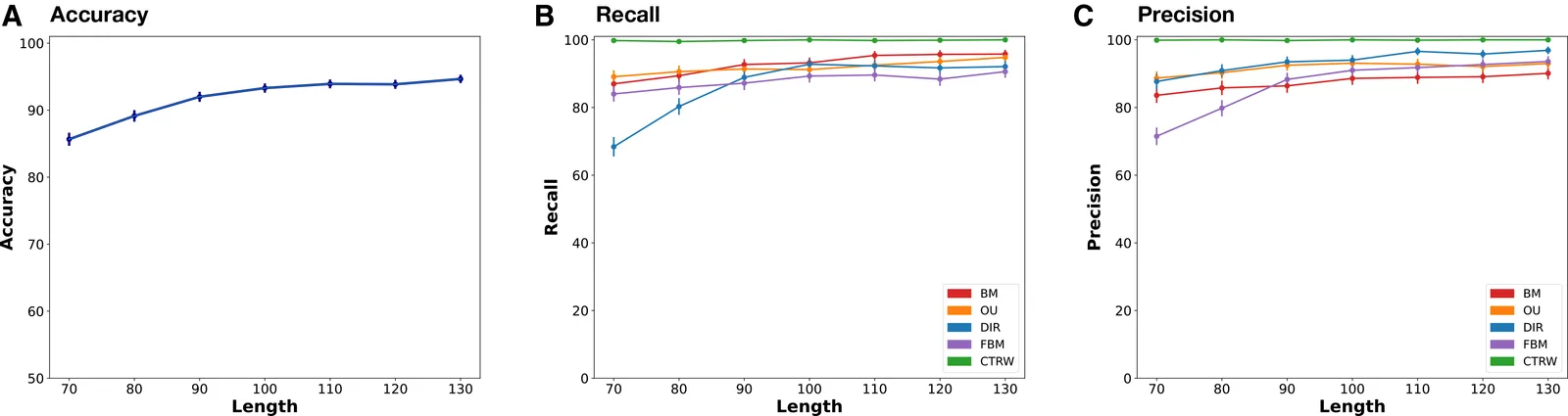
Classification performance under trajectory length mismatch between training and testing (A–C) (A) Overall accuracy, (B) recall, and (C) precision for each motion class (BM, OU, DIR, FBM, and CTRW) for a classifier trained on trajectories of length *N* = 100 and tested on trajectories with lengths ranging from *N* = 70 to *N* = 130. Error bars indicate 95% binomial proportion confidence intervals, computed over *M* = 10 independent simulations per trajectory length.

It is therefore essential to assess how discrepancies in trajectory length between training and testing data affect the model’s performance. Establishing an appropriate threshold for *N* can ease data collection and subsequent analysis. To investigate this, we trained the model on trajectories of length *N* = 100 and evaluated it on test datasets containing trajectories of lengths ranging from *N* = 70 to *N* = 130. The results are shown in Figure 7.

We observe that testing the model on shorter trajectories (*N* ≤ 90) results in a tendency to misclassify DIR paths as FBM, while FBM trajectories are most often misclassified as OU motion. BM is also sensitive to length mismatch, frequently being confused with the DIR class. In contrast, OU and CTRW trajectories remain well classified even when shorter lengths are used.

Interestingly, performance slightly improves when the model is tested on longer trajectories. Notably, when the trajectory length variation is limited to Δ*N* = 10 in the shorter direction (*N* > 90), the maximum drop in classification accuracy is only 1.6% compared to the baseline performance on *N* = 100 trajectories (93.3% accuracy; Figure 7). This demonstrates the robustness of our method to moderate variations in trajectory length (up to Δ*N* = 10), enabling more flexible data acquisition without sacrificing classification accuracy.

#### Comparison with state-of-the-art methods

To evaluate the effectiveness of the proposed method, we compare its performance against several state-of-the-art approaches for trajectory classification. Specifically, we selected methods that are widely used in the field and representative of distinct modeling paradigms, including null-hypothesis testing (maximum distance test [MDT]^7^), statistical fitting (divide-and-conquer MSS [DC-MSS]^31^), and deep learning (RANDI^40^). Detailed descriptions of these methods are provided in the STAR Methods.

To the best of our knowledge, no existing method in the literature is designed to distinguish the complete set of stochastic processes considered in this work. Therefore, for a fair comparison, we evaluate each method on the subset of motion types that are common between that method and our proposed classifier.

For the MDT, we consider three diffusion regimes: BM, subdiffusive motion (represented by FBM and CTRW), and superdiffusive motion (represented by DIR). For the DC-MSS method, which is designed to classify confined, BM, and DIR, the comparison is limited to BM, DIR, and confined motion, with the latter modeled in our framework by OU processes. For the RANDI model, which supports classification of FBM, CTRW, and other anomalous diffusion types, we restrict the evaluation to FBM and CTRW—the two classes common to both our classifier and the RANDI model’s training domain.

The comparison results are summarized in Table 5, which reports the recall values per motion class and the corresponding performance gap relative to our proposed method.

**Table 5:** Comparison with state-of-the-art methods

Method	BM	OU	DIR	FBM	CTRW
Max. dist. test^7^	95.4 ± 0.6	**95.8** ±**0.6**	–	80.0 ± 1.1	–
DC-MSS^31^	**95.5** ± **0.6**	61.7 ± 1.3	92.2 ± 0.7	–	–
RANDI^40^	–	–	–	**93.7** ± **0.7**	98.6 ± 0.3
Proposed	93.2 ± 0.7	91.2 ± 0.8	**92.8** ± **0.7**	89.3 ± 0.9	**100.0** ± **0.0**

For each method, we report the recall (%) for each motion class. “–” indicates that the method was not evaluated on the corresponding class. Values are reported as mean ± 95% binomial confidence interval (M = 10 simulations per trajectory length). Bold values correspond to the highest recall (%) obtained for each classified motion.

First, the results obtained with MDT show recall values for BM and OU that are comparable to those of our approach. However, the recall for FBM is reduced by 9%, with many FBM trajectories misclassified as BM. This performance drop highlights the limited robustness of the maximum distance criterion in distinguishing FBM from BM, particularly in the superdiffusive regime. This is essentially because distance-based criteria can distinguish only the diffusion nature of trajectories without giving information about their intrinsic dynamical properties. FBM paths with a Hurst parameter close to 1/2 can behave similarly to BM paths in terms of diffusion, which explains the results obtained using the MDT. On the other hand, our method introduces geometric features characterizing the dynamics beyond their diffusion properties, which enables highlighting FBM paths more accurately.

To apply the statistical fitting method DC-MSS to our dataset of trajectories with length *N* = 100, we computed the MSS slopes and applied the classification thresholds provided by Vega et al.^31^ (see STAR Methods). The results in Table 5 show good agreement with our method for the BM and DIR classes. In contrast, the performance for OU trajectories is significantly lower, suggesting that the fixed thresholds used in DC-MSS are not well suited for detecting OU-type confinement. This gap is essentially due to the class of confined motions used to tune the thresholds defined in the DC-MSS method. However, confined dynamics can be generated by distinct processes that cannot be discriminated using the same thresholds. This emphasizes the importance of moving beyond diffusion-level classification to a more detailed, process-based approach.

Finally, we benchmark our method against the deep learning-based RANDI classifier on the subset of motion classes common to both methods—FBM and CTRW. To this end, we retrained the RANDI model on the AnDi challenge dataset using trajectories of length *N* = 100 and evaluated it on our test dataset restricted to FBM and CTRW trajectories of the same length. The results in Table 5 indicate that RANDI achieves slightly higher accuracy for FBM classification but shows reduced performance on CTRW trajectories. While the overall accuracy across both classes is comparable to our method, it is worth noting that our feature-based approach offers interpretability, a significant advantage in biophysical applications.

The results demonstrate that our process-based classification method offers notable advantages over existing diffusion- and learning-based approaches. While MDT and DC-MSS provide reasonable accuracy for standard diffusion regimes such as BM and DIR, they struggle with more complex processes. Specifically, MDT fails to reliably distinguish FBM from BM and completely misclassifies CTRW trajectories, reflecting its limitations in capturing anomalous or non-ergodic dynamics. DC-MSS, although effective for DIR and BM, performs poorly on OU trajectories due to its reliance on fixed thresholds not tuned for such confined dynamics.

Compared to RANDI, our method achieves comparable overall accuracy on FBM and CTRW trajectories, with slightly lower performance on FBM but better classification of CTRW. Importantly, our approach provides interpretability through the use of geometric and statistical features—an asset that deep learning models like RANDI typically lack. This interpretability supports downstream analysis and enhances the applicability of our method in biophysical contexts.

Overall, the results demonstrate that our process-based classification framework not only performs well on classical diffusion regimes such as BM and DIR but also excels at identifying more complex stochastic processes, including OU and CTRW dynamics. Unlike diffusion-based methods, our approach captures finer distinctions between subdiffusive behaviors arising from different physical mechanisms—for example, differentiating FBM from OU or CTRW trajectories. It also distinguishes superdiffusive FBM from DIR, which is often a challenge for simpler classifiers. Notably, our method reliably identifies CTRW paths, which are frequently misclassified by existing techniques. In addition to its accuracy, the use of interpretable geometric and statistical features makes our approach particularly valuable for biophysical investigations, where insight into the underlying mechanism is essential. These strengths make the proposed method a robust and versatile tool for detailed trajectory classification across a range of diffusion behaviors.

### Characterizing CCR5 dynamics at the cell membrane

CCR5 is a membrane receptor that plays a significant role in inflammation and in HIV infection, acting as the major entry co-receptor. CCR5 can adopt several structural configurations, including different dimeric states, which interact differently with ligands or HIV envelopes and define subpopulations with distinct therapeutic potential. The study of CCR5 dynamics under different experimental conditions has been initiated in previous works^4^^,^^45^ to investigate how receptor motion relates to biological function in infection pathways. As these studies relied on diffusion-based classification schemes, similar experimental data can be reinterpreted within a process-based framework, enabling a more mechanistic description of receptor dynamics in terms of underlying stochastic models and highlighting the added value of our approach.

**Figure 8:**
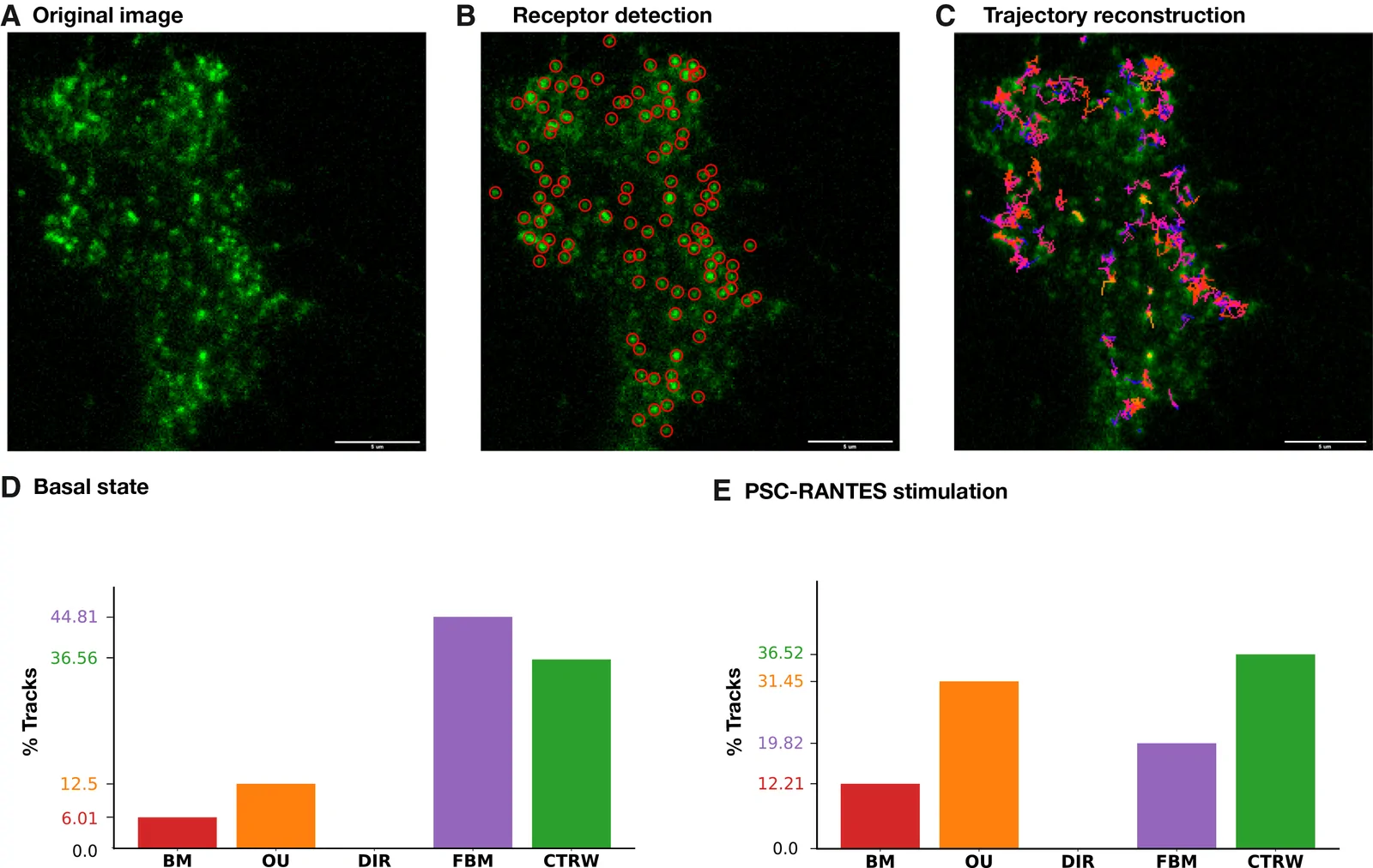
Analyzing CCR5 receptor dynamics by TIRF single-particle tracking (A) Time-lapse TIRF fluorescence image of GFP-CCR5 receptors at the plasma membrane. Scale bar, 5 m. (B) Automatic detection of individual fluorescent receptor spots (red circles) using the *spot detector* plugin of the Icy software.^48^^,^^49^ (C) Reconstruction of single-receptor trajectories using the *spot tracking* plugin^50^ of Icy (colored lines indicate individual trajectories). (D and E) Distribution of motion classes for CCR5 trajectories in the basal (unstimulated) condition (848 tracks, 16 cells) (E) Distribution of motion classes after stimulation with the superagonist PSC-RANTES (20 nM, 20 min 868 tracks, 43 cells). Trajectories were analyzed using segments of length *N* = 100 ± 10 with time step Δ*t* = 1/30 s. Motion classes BM, OU, DIR, FBM, and CTRW. Bar heights indicate the fraction of trajectories assigned to each class.

To this end, we performed time-lapse imaging of fluorescent CCR5 under various experimental conditions. Images were acquired using TIRFM, which enables visualization of a thin layer of the plasma membrane (approximately 200 nm). Videos were collected at 30 fps (consistent with the method using Δ*t* = 1/30) by imaging entire cells. The images were processed using the *spot detector* and *spot tracking* plugins of the Icy software,^48^ allowing detection of receptors and reconstruction of trajectories. Figure 8 illustrates the steps of the tracking analysis in Icy. Spots of size 3 pixels (300 nm) were detected, and an immobility threshold of 1 pixel was applied to each path to correct positions and highlight immobile receptors.

To track CCR5 along long trajectories without ambiguities, we used cells expressing low levels of CCR5 at the cell surface. Cells expressing CCR5 under the RUSH system (retention using selective hooks)^51^^,^^52^ were employed. This system allows synchronization and study of proteins following the biosynthesis/secretion pathway, based on intracellular retention and release upon biotin induction. Even without induction, RUSH-CCR5 cells express CCR5 at a low level due to leakage. The RUSH-CCR5 construct expresses CCR5 fused to GFP. Surface GFP-CCR5 was detected using a GFP booster (ATTO647N), overcoming background noise from receptors along the secretion pathway (see STAR Methods).

This technique enables imaging receptors in a low-density environment, facilitating detection and trajectory reconstruction, minimizing tracking errors, and allowing collection of longer trajectories. Based on the analysis of length inequality, the trained model for *N* = 100 can be applied to trajectories containing 90–120 time points, yielding a dataset of approximately 1,000 trajectories.

Motion classification was performed to compare basal-state receptors with those treated with PSC-RANTES, an analog of the natural chemokine RANTES/CCL5 with potent anti-HIV activity. PSC-RANTES inhibits infection by increasing CCR5 downregulation, acting as a superagonist that recognizes a broader array of CCR5 conformational states.^53^ Cells were incubated with or without 20 nM PSC-RANTES for 20 min, and receptor motion was tracked.

Results (Figure 8) reveal the coexistence of several motion subpopulations governed by distinct stochastic processes, indicating that CCR5 receptors experience heterogeneous local environments at the plasma membrane. In the basal condition, diffusion is not monolithic: rather than a single Brownian regime, trajectories are distributed across multiple dynamical classes. Upon PSC-RANTES stimulation, receptor dynamics are markedly altered, with a pronounced redistribution of trajectories across motion types. Overall, our analysis provides a robust and quantitative framework for motion classification, extending previous diffusion-based approaches,^4^^,^^45^ which reported predominantly Brownian basal dynamics and a transition toward subdiffusive behavior following PSC-RANTES treatment.

The present work reveals a more nuanced dynamical landscape. In the basal state, the majority of trajectories are classified as either intermittent motion (CTRW, 36.56%) or dynamics with long-range temporal correlations consistent with FBM (44.81%), whereas purely BM represents only a minor fraction (6.01%). FBM likely reflects generic receptor diffusion within the crowded plasma membrane, while CTRW may capture transient trapping events associated with entry into nanodomains or interactions with binding partners. Following 20 min of PSC-RANTES stimulation, the proportion of FBM trajectories decreases substantially to 19.82%, concomitant with an increase in OU-like dynamics (from 12.5% to 31.45%), indicative of receptor motion biased toward local equilibrium points. This redistribution highlights activated receptor motion landscape and is consistent with receptor clustering and internalization through clathrin-coated pits (CCPs) after 20 min of stimulation.^4^^,^^53^^,^^54^ A direct validation of this interpretation would require colocalization with membrane-associated markers (e.g., clathrin, G proteins, and *β*-arrestin) and super-resolution imaging to resolve receptor clustering at the single-molecule level.^55^ In conclusion, the proposed method turns out to be a powerful lens to finely capture the CCR5 dynamics, enabling the classification of different behaviors described by distinct stochastic laws and correlating with specific environmental constraints. Thus, this allows a deeper investigation of the dynamic life of receptors, opening the door to a more precise understanding of their functional activity.

## Discussion

We have presented a classification method designed to infer the stochastic processes (BM, OU, DIR, FBM, and CTRW) underlying particle trajectories. Specifically tailored to distinguish between different subdiffusive behaviors, the method is particularly well suited for capturing biophysical constraints encountered in cellular environments, such as trapping, confinement, and restricted mobility. It consistently refines broad diffusive categories and accurately detects non-ergodic dynamics, especially those described by CTRW.

A major advantage of our approach lies in its interpretability: the classification relies on geometric descriptors of trajectories within a supervised learning framework. This allows for a meaningful link between particle dynamics and their geometric signatures, enabling insights into how specific molecules modulate signaling through their impact on receptor mobility. As such, the method offers a new avenue for classifying particle dynamics with implications for understanding cellular signaling.

In addition to its strong performance on simulated data, the method demonstrates robustness to key challenges associated with experimental acquisition. Its classification accuracy remains stable under typical levels of localization noise, indicating that the selected features reflect intrinsic properties of the underlying stochastic processes.

We applied the method to characterize the dynamics of CCR5 membrane receptors, providing new insights into their behavior. We show that treatment with the agonist PSC-RANTES significantly alters receptor dynamics, inducing clear shifts in subdiffusive motion regimes. The observed shift in dynamical regimes upon PSC-RANTES stimulation likely reflects increased membrane confinement and CCR5 clustering within pre-endocytic domains, consistent with the early steps of clathrin-mediated internalization. Altogether, these results highlight the utility of process-based classification for uncovering functional effects of ligands and therapeutic agents on receptor motion, a critical step toward understanding and targeting signaling pathways.

We believe that integrating the proposed method with recent advances in live-cell imaging technologies^56^^,^^57^ will open new possibilities for studying the interplay between molecular dynamics and biophysical constraints in complex cellular environments.

### Limitations of the study

A first limitation of our approach is that it considers trajectories of fixed length, which requires using distinct models for datasets containing paths of different lengths. Nevertheless, the method demonstrates resilience to variations in trajectory length between training and testing data, maintaining high accuracy even when trajectories differ by up to 10% in length. This robustness makes the method practical for real-world experimental workflows, where trajectory lengths often vary due to tracking limitations or biological variability.

A second important consideration is the accuracy of reconstructed trajectories used as input. While issues related to tracking errors are beyond the scope of this work, we identified several experimental factors that could affect trajectory quality. For example, particle density within the imaged domain can complicate distinguishing individual trajectories during long-term tracking, potentially leading to mixed paths. Additionally, variability in particle fluorescence can affect local detection, sometimes causing trajectory splitting. To mitigate these challenges, we employed specific experimental techniques (RUSH-CCR5) that control receptor expression levels at the membrane. This approach allows imaging in a low-density environment with stabilized fluorescence signals, minimizing tracking errors and enabling the collection of longer, more reliable trajectories.

The present study focuses on a set of canonical stochastic processes commonly used to describe particle motion. In practice, however, many biological and physical systems may exhibit continuously varying dynamical parameters or gradual transitions between distinct stochastic regimes. For example, the Hurst exponent of an FBM may evolve continuously in response to changes in the local environment, while receptors may alternate between free diffusion, confinement, and directed transport along a single trajectory. Extending the proposed framework to characterize trajectories along such continua, for instance by tracking variations in the Hurst exponent or by detecting transitions between motion regimes through time-resolved analyses, constitutes an interesting avenue for future work.

More generally, some trajectories may display heterogeneous dynamics across multiple spatial and temporal scales, making them difficult to describe using a single stationary process. In this context, alternative models such as Lévy flights or Lévy walks may provide a useful framework for capturing intermittent long-range displacements interspersed with periods of local exploration. Integrating such multiscale stochastic processes into the present classification framework could provide a more refined description of non-stationary and heterogeneous dynamics, while helping bridge the gap between discrete process classification and the continuous spectrum of behaviors observed in experimental trajectories.

## Resource availability

### Lead contact

Requests for further information and resources should be directed to and will be fulfilled by the lead contact, Thibault Lagache (thibault.lagache@pasteur.fr).

### Materials availability

This study did not generate new materials.

### Data and code availability

• All data reported in this paper will be shared by the lead contact upon reasonable request.
• The source code used for trajectory simulation, feature extraction, and motion classification is publicly available at: https://gitlab.pasteur.fr/bia/projects/stochastic-dynamics-classification
• Any additional information required to reanalyze the data reported in this paper is available from the lead contact upon reasonable request.

## Acknowledgments

This work was funded by the 10.13039/501100001665ANR via grant nos. ANR-10-LABX-62-IBEID (LabEx IBEID), ANR-16-CONV-0005 (PIA INCEPTION), ANR-21-CE44-0030 03 (GET-REDI), and ANR-10-INBS-04 (France BioImaging) and ANRS (AAP 2025-1 -ECTZ325296).

## Author contributions

Conceptualization, G.N., M.S.S., J.-C.O.-M., and T.L.; methodology, G.N. and M.S.S.; investigation, G.N. and M.S.S.; data acquisition, A.B. and M.B.; data analysis, G.N.; writing – original draft, G.N.; writing – review and editing, G.N., A.B., J.-C.O.-M., and T.L.; funding acquisition, A.B., J.-C.O.-M., and T.L.; resources, J.-C.O.-M. and T.L.; supervision, J.-C.O.-M. and T.L.

## Declaration of interests

The authors declare no competing interests.

## STAR★Methods

### Key resources table

**Table:** 

REAGENT or RESOURCE	SOURCE	IDENTIFIER
**Chemicals, peptides, and recombinant proteins**		

PSC-RANTES	NIBSC (Center for AIDS Reagents)	N/A
GFP Booster ATTO647N	Proteintech	Cat# gba647n; RRID: AB_2629215

**Experimental models: Cell lines**		

Human: HeLa RUSH-CCR5-WT cells	Gift from G. Boncompain's lab	N/A

**Software and algorithms**		

Icy	https://icy.bioimageanalysis.org	Icy
Python	https://www.python.org	Python 3
scikit-learn	https://scikit-learn.org	N/A
FBM Python package	https://pypi.org/project/fbm/	N/A
Trajectory classification code	https://gitlab.pasteur.fr/bia/projects/stochastic-dynamics-classification	N/A

### Experimental model and study participant details

#### Cell line

HeLa cells stably expressing streptavidin-KDEL_SBP_-EGFP-tagged CCR5-WT (RUSH-CCR5-WT)^52^ were used throughout this study. Cells were cultured in Dulbecco’s Modified Eagle Medium (DMEM, Thermo Fisher Scientific) supplemented with 10% fetal bovine serum (FBS, Life Technologies) and 100 *μ*g/mL penicillin–streptomycin at 37°C in a humidified atmosphere containing 5% CO_2_.

Cell lines were not specifically tested for mycoplasma contamination for this study. Cell line authentication was not performed, as the stable cell line was generated and maintained in-house. Because this study focused on receptor trafficking and single-particle dynamics in an immortalized cell line, the sex of the original donor was not considered as an experimental variable and is not expected to influence the conclusions of this work.

### Method details

The proposed framework consists of four successive stages (Figure 1). First, synthetic trajectories are generated from five stochastic motion models to construct the datasets used for training and validation. Second, four interpretable geometric features describing trajectory directionality and spatial spreading are extracted. Third, these features are used to train a Random Forest classifier that assigns each trajectory to its underlying stochastic process. Finally, the trained classifier is applied to experimental single-particle trajectories acquired by TIRF microscopy.

#### Simulating discrete stochastic dynamics

To generate training and validation datasets with known ground truth, we simulated trajectories from five stochastic processes representing the principal classes of membrane receptor dynamics observed experimentally: 1) free motion characterized by BM; 2) confined and local movements generated by the OU process; 3) DIR governed by a drift; 4) constrained evolutions due to a crowded and viscous cellular medium described by FBM; 5) movements alternating jumps and pauses describing trapping phenomena and modeled by CTRW.

These five stochastic laws can be described theoretically using stochastic differential equations. The discretization of the related equations enables thus the simulation of discrete trajectories used for machine-learning method training and validation. A complete presentation of the mathematical properties of the considered processes, and a description of their numerical simulation is provided in supplemental information. These datasets are used in the rest of the paper for illustrating the introduced features, and for method training and validation. Consistently with standard fluorescence microscopy acquisitions considered in this work, simulations are performed in two dimensions, using micrometric (*μm*) displacements and a time interval of 1/30*s* between successive trajectory positions, corresponding to 30 Hz imaging acquisition.

Simulated trajectories exhibit the dynamic behavior induced by the related process (e.g., confinement, trapping, constrained evolutions). This results in trajectories with characteristic spatial evolutions whose geometric properties allow inferring the corresponding process. This justifies the features-based approach used in this work, which is based on geometrical descriptors of trajectories encoding the different dynamic characters and enabling process discrimination.

#### Designing trajectory geometric features

The proposed classifier relies on four interpretable geometric features. Two quantify trajectory directionality, whereas two characterize trajectory spreading. Together, these descriptors capture complementary aspects of stochastic particle dynamics (Figure 1C).

*Directionality* can be described using the distribution of angles between successive positions, encoding the persistent, Brownian, or anti-persistent character of trajectories.^28^ This translates into our framework identifying paths driven by restoring forces (majority of large angles, corresponding to OU or FBM (*H* < 1/2)), propagation behavior (majority of small angles, corresponding to DIR or FBM (*H* > 1/2)) and random motion (BM is characterized by uniform-like angle distribution). On the other hand, due to trapping, most of the angles between successive positions are null in CTRW paths.

The second set of features describes the *spreading* properties of particles based on Ripley’s indices on concentric balls (see Equation 6). This allows describing how the particle unfurls in space and helps, in particular, to depict confined (OU) and spreading (DIR) motions. To our knowledge, this work is the first to use this family of features for motion characterization.

A discrete trajectory is given by a set of *N* positions $X_{i}\in R^{2}$, with independent components, corresponding to different times $t_{1}<\ldots <t_{N}\in R^{+}$:

(Equation 2) $$X=\left(X_{t_{1}},\ldots ,X_{t_{N}}\right).$$

##### Description of trajectory directionality

For every three consecutive points of the trajectory $\left(X_{t_{i}},X_{t_{i+1}},X_{t_{i+2}}\right)$, we consider the angle^28^:

(Equation 3) $$\theta_{X}\left(t_{i}\right)=\pi -∠X_{t_{i}}X_{t_{i+1}}X_{t_{i+2}},$$

which is considered as positive if $X_{t_{i+1}}-X_{t_{i}}$ rotates on $X_{t_{i+2}}-X_{t_{i}}$ in a counterclockwise way. In the case of CTRW, most angles are null since the particle often remains stationary at the same point. Therefore, non-null angles are computed between triplets of not necessarily consecutive displacements.

As shown in Figure 2, the density $p_{\theta_{X}}$ of angle distribution exhibits a different analytical dependence for different processes. BM has a uniform distribution of angles because of its random displacements.^28^ On the other hand, a motion governed by a drift exhibits displacements around the privileged direction resulting in small angles between successive positions; then, the histogram of the angles can be approximated by a Gaussian distribution centered at zero. On the other hand, in confined motions driven by restoring forces, most of the angles must be larger than *π*/2 allowing the particle to go back to the equilibrium point. This suggests that the convexity of the angle distribution gives a first approximation of the histogram shape allowing for distinguishing different dynamics.

Thus, to estimate the **convexity of the angle histogram**, the following fitting is performed:

(Equation 4) $$p_{\theta_{X}}\left(x\right)\propto ax^{2},$$

and the value of *a* defines a geometrical feature describing the angle variability.

Moreover, to establish the existence of a preferred motion direction along the trajectory we also consider the following **index of directionality**^28^:

(Equation 5) $$P_{d}\left(X\right)=P\left(|\theta_{X}|<\pi /2\right)-P\left(|\theta_{X}|\geq \pi /2\right).$$

A positive *P*_*d*_ indicates that most of the angles are smaller than *π*/2 resulting in a persistent movement along a privileged direction (corresponding to diffusive dynamics). In contrast, a negative *P*_*d*_ indicates an anti-persistent movement exhibiting larger angles (corresponding to a confined dynamic governed by restoring forces).

##### Encoding trajectory spreading

The spreading character of a trajectory can be described by analyzing its displacements through concentric balls. This enables capturing (local) confinement or constant drift and quantifying the motion amplitude.

To this goal, we consider the Ripley’s index *K*_*r*_ in a ball $B\left(X_{t_{1}},r\right)$ of radius *r* centered at the starting point $X_{t_{1}}$ of the trajectory^58^:

(Equation 6) $$K_{r}=|\left\{X_{t_{i}}\in X\;|\;X_{t_{i}}\in B\left(X_{t_{1}},r\right)\right\}|/N,$$

accounting for the number of trajectory points living in the ball $B\left(X_{t_{1}},r\right)$.

To make this computation consistent with the trajectory dynamics, we define the reference radius:

(Equation 7) $$R=\frac{1}{N}\overset{N-1}{\underset{i=1}{\sum }}\|X_{t_{i+1}}-X_{t_{i}}\|$$

which corresponds to the mean displacement between consecutive points of the trajectory, and we compute the Ripley vector

(Equation 8) $$K_{X}=\left(K_{R},\ldots ,K_{kR},\ldots ,K_{NR}\right),$$

with 1 ≤ *k* ≤ *N*. The reference radius *R* is computed individually for each trajectory to standardize the analysis relative to the parameters of the corresponding stochastic motion. Rather than focusing on absolute spreading, we aim to capture the dynamics of spreading. For instance, using a fixed radius for all paths could result in similar absolute spreading and *K*_**X**_ curves for DIR trajectories with small parameters ‖*u*‖, *σ* and OU trajectories with larger parameters *λ*, *σ*. However, we expect *K*_**X**_ to reach a plateau quickly for OU paths, while increasing more slowly for DIR paths. By selecting a trajectory-dependent *R*, defined as the mean displacement between successive positions, we emphasize these intrinsic differences in spreading behavior.

Figure 5 presents examples of the vectors *K*_**X**_ for various simulated processes. Distinct profiles emerge, with processes such as OU, BM, and FBM reaching their maximum value *K*_**X**_ = 1 more rapidly than CTRW and DIR. In particular, the CTRW process exhibits local plateaus in the *K*_**X**_ curve, reflecting periods of inactivity or pauses in the trajectory. These differences indicate that the analytical form of Ripley’s curve *K*_**X**_ can serve as an effective descriptor to distinguish between different dynamical behaviors.

*K*_**X**_ is a non-decreasing function that satisfies *K*_*kR*_ = 1 for all *k* ≥ *k*_0_ ∈ [1, *N*], where *k*_0_*R* exceeds the maximal distance traveled by the particle from its initial position. This motivates estimating the analytical form of the **Ripley curve** *K*_**X**_ as a function of the index *k*, using the following exponential fit:

(Equation 9) $$K_{X}\propto 1-e^{-bk}.$$

We then consider the parameter *b* as a third feature of interest. Figure 4A displays the values of *b* for a set of trajectories simulated under different processes. Larger values of *b* are observed for subdiffusive dynamics such as OU and FBM, which quickly reach their maximal displacement. In contrast, superdiffusive processes like DIR exhibit smaller values of *b*, reflecting a more gradual increase in *K*_**X**_ as the particle explores space along a preferred direction.

Finally, we also consider the proportion of points in *K*_**X**_ with a non-zero discrete derivative $K_{X}^{\prime }$:

(Equation 10) $$P_{p}\left(X\right)=P\left(K_{X}^{\prime }\neq 0\right),$$

where $K_{X}^{\prime }$ denotes the discrete derivative of *K*_**X**_ with respect to index *k*:

(Equation 11) $${\left(K_{X}^{\prime }\right)}_{kR}={\left(K_{X}\right)}_{\left(k+1\right)R}-{\left(K_{X}\right)}_{kR},$$

for *k* = 1, …, *N* − 1.

A zero derivative corresponds to local plateaus in the Ripley curve—referred to as **Ripley’s plateaus**—which arise when the Ripley index remains constant across successive concentric balls. These plateaus indicate transient local confinement in the particle’s movement.

Figure 4B shows the values of *P*_*p*_(**X**) for simulated trajectories generated by various processes. Low values of *P*_*p*_(**X**)—i.e., a high proportion of points with zero derivative—are observed for OU, subdiffusive FBM (with Hurst coefficient $H\ll \frac{1}{2}$), CTRW models that incorporate pauses. In contrast, high values of *P*_*p*_(**X**) correspond to spreading motions, characterized by a persistent directional component and limited backward movement, as seen in superdiffusive processes such as DIR and FBM with $H\gg \frac{1}{2}$.

The observed ranges of the fitting parameter *b* and the proportion *P*_*p*_(**X**) of non-zero derivatives across different simulated processes (Figure 4) highlight how these two metrics can discriminate between various spreading behaviors. As such, they serve as useful features for classifying stochastic motions within a machine-learning framework.

#### Classification method

The objective of the classification stage is to infer the stochastic process underlying each trajectory from the four geometric descriptors introduced above. We selected a Random Forest classifier because it combines high predictive performance with straightforward interpretability through explicit feature representations and feature importance analysis.

Training and validation are performed on independent datasets generated according to the procedures previously described, using the process labels—BM, OU, DIR, FBM, and CTRW—as classification targets. We employ the Random Forest classifier from the *scikit-learn* Python package, using the default setting of 10 estimators. The model is trained and validated on datasets comprising 5000 trajectories, with 1000 samples per motion type.

Compared to standard classification algorithms such as *k*-nearest neighbors (k-NN) or support vector machines (SVM), the Random Forest classifier excels in capturing complex, non-linear relationships within the data. It aggregates predictions from an ensemble of decision trees (ten in our implementation), without requiring any *a priori* assumptions on the data space structure. This leads to a robust classification framework with minimal hyperparameter tuning, while still providing interpretability and the ability to assess feature importance.

#### Simulating stochastic trajectories

Training and validation datasets were generated by simulating independent trajectories from the five stochastic processes considered in this study. Unless otherwise specified, each dataset contained 1000 trajectories per motion class for a fixed trajectory length *N*.

The temporal sampling interval Δ*t* determines the discretization of the underlying continuous stochastic process and directly affects the statistical properties of trajectory increments (e.g., the variance and covariance of BM, FBM, and OU trajectories). Since the proposed framework is intended for the analysis of molecular trajectories acquired by two-dimensional fluorescence microscopy, simulations were designed to reproduce the spatial and temporal resolutions typically encountered in confocal and total internal reflection fluorescence microscopy (TIRFM). Throughout this work, the sampling interval was fixed to Δ*t* = 1/30 s, corresponding to an acquisition frequency of 30 Hz. Varying Δ*t* changes the temporal resolution at which the stochastic process is observed and may alter the extracted geometric features through under- or oversampling. The influence of the acquisition frequency was therefore not investigated here, as the objective was to evaluate classification performance under a fixed experimental imaging protocol.

Brownian motion (BM) trajectories were generated by iteratively sampling independent Gaussian increments with diffusion parameter *σ* uniformly distributed in the interval [0.1, 10].

Ornstein–Uhlenbeck (OU) trajectories were generated with *σ* ∈ [1, 10]. For each trajectory, the restoring coefficient was defined as

$$\lambda =\frac{r\sigma }{\sqrt{\Delta t}},$$

where the dimensionless ratio *r* was sampled uniformly in [0.2, 1]. Trajectories were generated using the *DiffusionProcess* class of the Python package *stochastic*.^59^

Directed (DIR) trajectories were generated by iteratively adding a Gaussian increment and a constant drift vector. The diffusion parameter *σ* was uniformly sampled in [1, 10], while the drift direction was fixed to (1, 1). The drift vector was therefore defined as

$$\mu =\frac{r\sigma }{\sqrt{2\Delta t}}\left(1,1\right),$$

so that its Euclidean norm satisfies

$$\|\mu \|=\frac{r\sigma }{\sqrt{\Delta t}},$$

where *r* is uniformly sampled in [0.2, 1].

The normalization by $1/\sqrt{\Delta t}$ in the OU and DIR models ensures that the parameter *r* represents the relative contribution of deterministic and stochastic increments independently of the temporal sampling interval.

Fractional Brownian motion (FBM) trajectories were simulated using the Hosking algorithm,^60^ implemented in the Python package *FBM*.^61^ The Hurst exponent was sampled uniformly from the intervals [0.15, 0.30] ∪ [0.70, 0.85], corresponding to strongly subdiffusive and strongly superdiffusive dynamics, respectively. Values close to *H* = 1/2 were deliberately excluded in order to avoid trajectories whose dynamics are nearly indistinguishable from classical Brownian motion.

Continuous-time random walk (CTRW) trajectories were generated using Gaussian jump lengths with *σ* uniformly sampled in [0.1, 10]. Waiting times were sampled from an exponential distribution with parameter *γ* ∈ (0, 1) and truncated to the interval [1, *N*/5] to avoid waiting times exceeding the characteristic duration of the simulated trajectories. Jumps and waiting periods were generated iteratively until the cumulative waiting time exceeded the trajectory duration, after which the trajectory was truncated to *N* time points.^62^

The simulation parameters used for each stochastic process are summarized in Table 1.

#### State-of-the-art methods considered for comparison

To benchmark the proposed framework, we compared its performance with three representative approaches for trajectory classification based on statistical hypothesis testing, moment scaling analysis, and deep learning, respectively.

##### Maximum Distance Test (MDT)

The Maximum Distance^7^ Test performs diffusion-state classification using a statistical hypothesis test on trajectories of fixed length. Unlike approaches relying on mean squared displacement (MSD), which are known to be unreliable for short trajectories, MDT uses the standardized maximal displacement from the initial position,

$$T_{N}=\frac{\max_{k=0,\ldots ,N}\|X_{t_{k}}-X_{t_{0}}\|}{{\left[\frac{1}{2}\overset{N}{\underset{i=1}{\sum }}\|X_{t_{i}}-X_{t_{i-1}}{\|}^{2}\right]}^{1/2}}.$$

The empirical 2.5% and 97.5% quantiles of *T*_*N*_ computed from Brownian trajectories define the following three-class statistical test:

(Equation 12) $$Class=\left\{\begin{array}{cc} \text{subdiffusive}, & T_{N}<q_{2.5},\\ \text{superdiffusive}, & T_{N}>q_{97.5},\\ \text{Brownian}, & \text{otherwise}. \end{array}\right)$$

For comparison with our method, subdiffusive trajectories correspond to FBM and CTRW, whereas superdiffusive trajectories correspond to DIR.

##### Divide-and-Conquer Moment Scaling Spectrum (DC-MSS)

DC-MSS characterizes^31^ particle motion through the Moment Scaling Spectrum (MSS), which generalizes the mean squared displacement to arbitrary statistical moments. For a time lag *τ*, the generalized moment of order *p* is defined as

$$M_{p}\left(\tau \right)=\frac{1}{N-\tau }\overset{N-\tau }{\underset{k=1}{\sum }}\|X_{t_{k+\tau }}-X_{t_{k}}{\|}^{p}.$$

The dependence of *M*_*p*_(*τ*) on the lag time is approximated by a power law,

$$M_{p}\left(\tau \right)\propto \tau^{\nu_{p}},$$

and the linear regression of *ν*_*p*_ as a function of *p* defines the MSS slope *S*_MSS_. Brownian trajectories are characterized by *S*_MSS_ ≈ 0.5, whereas smaller and larger values indicate confined and directed motion, respectively. Following,^31^ classification thresholds were taken directly from the original publication without additional optimization.

##### RANDI

RANDI is a recurrent^40^ neural network (RNN)-based classifier developed within the framework of the Anomalous Diffusion (AnDi) Challenge.^8^ Rather than relying on handcrafted descriptors, RANDI learns discriminative representations directly from raw two-dimensional trajectories. The original model was trained to recognize several anomalous diffusion processes, including FBM, CTRW, annealed transient time motion, Lévy walks, and scaled Brownian motion. For a fair comparison with our framework, the published implementation was retrained on trajectories of length *N* = 100 and evaluated only on the motion classes common to both methods (FBM and CTRW).

#### Experimental procedures

##### Reagents

HeLa cells stably expressing streptavidin-KDEL_SBP-EGFP tagged-CCR5-WT (referred to as RUSH-CCR5-WT)^52^ were maintained in Dulbecco’s Modified Eagle Medium (DMEM) (Thermo Fisher Scientific) supplemented with 10% fetal bovine serum (FBS, Life Technologies) and 100 *μ*g/mL penicillin/streptomycin (Life Technologies).

The chemokine analog PSC-RANTES was obtained through the Center for AIDS Reagents, National Institute for Biological Standards and Control (NIBSC, UK) (full chemical name: N-*α*-(n-nonanoyl)-des-Ser(1)-[L-thioprolyl(2), L-cyclohexylglycyl(3)] RANTES(4–68)).

The GFP booster ATTO647N was obtained from Proteintech.

##### Cell labeling

Round 25 mm No. 01 glass coverslips (Fisher Scientific) were pre-cleaned with absolute ethanol followed by acetone, with three consecutive washes in ddH_2_O. Cells (2.5 × 10^5^), plated onto pre-cleaned coverslips 48 h before imaging, were incubated with the GFP booster ATTO647N (1:500 dilution) for 5 min and washed ten consecutive times with TIRF medium (25 mM HEPES, 135 mM NaCl, 5 mM KCl, 1.8 mM CaCl_2_, 0.4 mM MgCl_2_, 4.5 g/L glucose and 0.5% BSA, pH 7.4). Cells were imaged in TIRF medium.

For the stimulated condition, cells were incubated at 37°C for 20 min with PSC-RANTES (20 nM) after staining with the GFP booster.

##### Live-cell TIRF imaging

Movies were acquired using an Elyra 7 microscope (Zeiss) equipped with a sCMOS camera (PCO Edge 4.2) and an *α* Plan-Apochromat 63×/1.46 oil objective, a 642 nm (500 mW) laser line, and a quad-band filter coupled to an LP 560 filter. Image acquisition was performed at 1 frame per 33 ms (30 Hz) for 400 frames, at 37°C and under a 5% CO_2_-enriched atmosphere. Under these conditions, spot intensity remained stable throughout acquisition.

### Quantification and statistical analysis

Trajectory feature extraction, model training, and performance evaluation were implemented in Python 3 using custom scripts. Random Forest classification was performed using the *scikit-learn* library with 10 decision trees and default hyperparameters unless otherwise specified.

Classification performance was evaluated using overall accuracy, recall, and precision. For simulated datasets, each experiment was repeated independently *M* = 10 times using newly generated trajectories. Reported values correspond to the mean over these independent realizations, and error bars represent two-sided 95% binomial confidence intervals.

Feature importance was assessed using a permutation-based mean decrease in accuracy (MDA) analysis. Each feature was randomly permuted while leaving the remaining features unchanged, and the corresponding decrease in recall was measured. The permutation procedure was repeated ten times for each feature.

Visualization of the four-dimensional feature space was performed using three-dimensional t-distributed stochastic neighbor embedding (t-SNE) implemented in the *scikit-learn* package.

For experimental data, receptor trajectories were reconstructed using the *Spot Detector* and *Spot Tracking* plugins of Icy. Based on the trajectory-length robustness analysis, only trajectories containing 100 ± 10 positions were retained for classification. Motion class proportions were computed from all classified trajectories for each experimental condition.

## Supplemental information

Document S1. Figure S1 and Table S1

Data S1. Source code implementing the stochastic trajectory simulation, geometrical feature extraction, and random forest classification pipeline used in this study
