# Supplementary material for: Characterizing cellular receptor dynamics in live fluorescence imaging using stochastic modeling and machine learning
Source: iScience. 2026 Sep 4;29(9):117435. doi: 10.1016/j.isci.2026.117435 (PMC13571939; doi:10.1016/j.isci.2026.117435)
Supplement: Supplementary file 1 [file mmc1.pdf]

iScience, Volume *29*

Supplemental information

Characterizing cellular receptor dynamics in live fluorescence imaging using stochastic modeling and machine learning

Giacomo Nardi, Matheus Santos Sano, Margaux Bilay, Anne Brelot, Jean-Christophe Olivo-Marin, and Thibault Lagache

Supplemental information

Modeling stochastic dynamics

General definitions. A stochastic process is a one-parameter family X_t of $\mathbb{R}^2$-valued random variables defined on a probability space $(\Omega, \mathcal{F}, \mathbb{P})$:

$$X_t \,:\, (t, \omega) \in \mathbb{R}^+ \times \Omega \mapsto X_t(\omega) \in \mathbb{R}^2 \,.$$

In particle tracking, the two coordinates of X_t are assumed to be independent random variables. The parameter t corresponds to the time and, for every $\omega \in \Omega$, the related trajectory is defined by the application :

$$t \in \mathbb{R}^+ \mapsto X_t(\omega) \in \mathbb{R}^2 \,.$$

The statistical properties of increments determine the main properties of stochastic processes. A process is said to have *stationary increments* if increments distribution only depends on the time interval and not on the current times (e.g., $X_t - X_s$ and $X_{t-s} - X_0$ follow the same probability distribution for every $s < t \in \mathbb{R}^+$). Similarly, a process is said to be *stationary* if the distribution of X_t does not depend on t. On the other hand, we say that a process has *independent increments* if $X_t - X_s$ and $X_s - X_w$ are independent random variables for every $w < s < t \in \mathbb{R}^+$, implying that each displacement is not correlated to previous realizations. In the following, we consider Gaussian processes, meaning the increments follow a Gaussian distribution. Finally, we recall that a process is said to be *continuous* if it has continuous trajectories $t \mapsto X_t$ with probability one on Ω :

$$\mathbb{P}(\{\omega \in \Omega \,|\, \lim_{s \to t} |X_s(\omega) - X_t(\omega)| = 0 \quad \forall t \in \mathbb{R}^+\} \,) = 1 \,.$$

The discrete version of a stochastic process can be obtained by sampling the continuous paths on a finite set of times $t_0, ..., t_N \in \mathbb{R}^+$, defining a *discrete-time stochastic process*. A discrete trajectory is defined as a set of successive positions over time

$$\mathbf{X} = (X_{t_1}, ..., X_{t_N}) \,,$$

where $X_{t_i} \in \mathbb{R}^2$ $(i = 1, ..., N)$ with independent components and the time interval between successive positions is constant.

MSD criterion. The MSD function estimates the relationship between the average of increments at a fixed time lag and the corresponding time interval. Since Einstein's seminal work on BM[1], the MSD has been used to distinguish Brownian dynamics from subdiffusive and superdiffusive dynamics[2,3], thereby enabling trajectory classification into three classes based on particle diffusion. However, as discussed below, this criterion is inadequate for a more precise analysis aiming at distinguishing different subdiffusive dynamics or detecting non-ergodic behaviors.

The MSD definition involves the ensemble average computed using the mean in the probability space[4]:

$$\langle X_t^2 \rangle_{ens} \,=\, \mathbb{E}(\|X_t - X_0\|^2)$$

This definition can be used whenever all the particles follow the same stochastic process. Then, each trajectory represents an independent realization of it, enabling the ensemble average in the probability space, computed on the displacements of all trajectories during the time lag t. However, in many biological contexts, particles can be governed by different processes and several dynamical behaviors can co-exist within the observed trajectories. In this case, the previous MSD definition is inadequate, and each trajectory needs to be analyzed individually. Then,

a time-averaged version of the MSD function is introduced, quantifying increments average as a function of the time interval for each trajectory X_t:

$$\langle X_t^2 \rangle_{T,\Delta t} = \frac{1}{T-\Delta t}\int_0^{T-\Delta t} \|X_{\tau+\Delta t} - X_\tau\|^2 \, d\tau \, . \tag{1}$$

For computational applications, (1) can be discretized as follows :

$$\text{MSD}_{time}(X_t, \Delta t) = \frac{1}{N-\Delta t+1}\sum_{k=1}^{N-\Delta t} \|X_{t_k+\Delta t} - X_{t_k}\|^2 \, , \tag{2}$$

where N is the number of positions belonging to the trajectory $\mathbf{X}$ and Δt is an integer.

In single-particle analysis, trajectories are classified based on the optimal power α for the fitting of the time-average MSD (2) to the generic polynomial $t \mapsto Ct^{\alpha}$. This enables the definition of the following MSD criterion for diffusion classification :

$$\text{Diffusion class} = \begin{cases} \text{subdiffusive}, & \text{if } \alpha < 1 \\ \text{free motion}, & \text{if } \alpha = 1 \\ \text{superdiffusive}, & \text{if } \alpha > 1 \end{cases} \tag{3}$$

These three classes are based on particle diffusion and physically represent confined, Brownian, and spreading motions (respectively). However, this is often an inadequate and limited framework for many biological applications, for the reasons discussed below.

First, a precise statistical estimate of the MSD coefficient needs long trajectories, which are often unavailable in single-particle tracking because of missed detection or temporal exit from the field of view. Moreover, in the case of BM, a tolerance threshold is needed to estimate the fitting coefficient $\alpha = 1$, which introduces a new parameter to tune in the method[5].

Secondly, in the case $\alpha < 1$, the MSD criterion detects subdiffusion, but it does not give any information about the intrinsic behavior of the trajectory. As detailed below, subdiffusive processes can reveal different dynamics (e.g., trapping, confinement) and a more precise classification of subdiffusive behaviors is required. Moreover, beyond identifying the process governing the trajectory, estimating trajectory's geometrical parameters is necessary to provide useful information on how the particle unfurls in space (for instance, privileged directions, stopping times, or spreading radius).

The last critical point about the MSD criterion concerns its consistency with ergodicity. In ergodic dynamical systems, particles visit every point of the space uniformly and randomly. This means that the statistical properties of the system can be estimated from typical trajectories rather than ensemble averages. The ergodic theorem[6,7] states that for an ergodic process, $\langle X_t^2 \rangle_{ens}$ and $\langle X_t^2 \rangle_{T,\Delta t}$ exhibit the same functional dependence on t and Δt, respectively. This implies that the MSD criterion based on time-average gives a result consistent with the ensemble-average estimate. However, for non-ergodic processes, time and ensemble-average MSD can exhibit different dependencies on time, making the MSD criterion inadequate to classify trajectories governed by non-ergodic processes. This is the case, for example, of CTRW detailed in the next section.

BM BM (also called free or normal motion in some works) is a continuous Gaussian stochastic process introduced in the seminal Einstein's work[1] on particle diffusion and widely used to model random motion. It is denoted by B_t, it starts at zero and has stationary and independent increments verifying :

$$(B_t - B_s) \sim \mathcal{N}(0, (t-s)\mathbf{I}_2)$$

for every $t > s$, where $\mathbf{I}_2$ denotes the 2-dimensional identity matrix. However, this process is not stationary as B_t follows a Gaussian distribution with a variance depending on t. Setting $X_t = \sigma B_t$ we can vary the displacement amplitude by a parameter σ, and we get

$$\langle X_t^2 \rangle_{ens} = 2\sigma^2 t\,, \quad \langle X_t^2 \rangle_{T,\Delta t} = 2\sigma^2 \Delta t$$

that justifies the introduction of MSD criterion.

DIR The main model for super-diffusion, which describes particles driven by active motors[8] verifies

$$dX_t = \mu dt + \sigma dB_t$$

where the drift component $\mu \in \mathbb{R}^2$ gives a constantly oriented input to the motion. Depending on the ratio $\|\mu\|/\sigma$, the trajectory can exhibits a spreading behavior along the direction μ ($\|\mu\| >> \sigma$) or a Brownian dynamics ($\|\mu\| << \sigma$). Similarly to BM, X_t is a non-stationary continuous Gaussian process with stationary and independent increments.

As the mean of B_t equals zero, a direct computation gives

$$\langle X_t^2 \rangle_{ens} = \|\mu\|^2 t^2 + 2\sigma^2 t\,, \quad \langle X_t^2 \rangle_{T,\Delta t} = \|\mu\|^2 (\Delta t)^2 + 2\sigma^2 \Delta t$$

showing the superdiffusive behavior of X_t according to the MSD criterion.

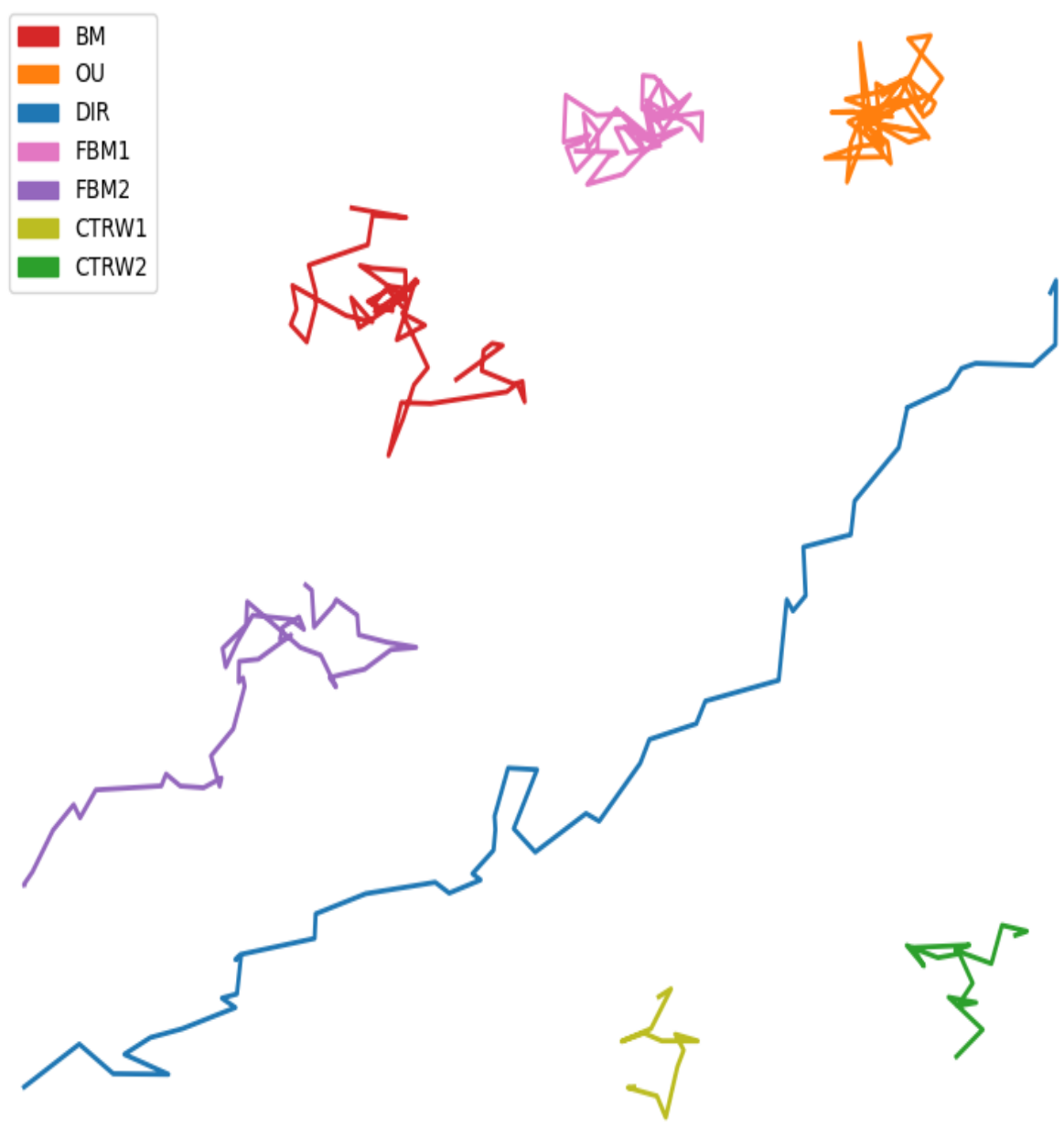

Figure S1: **Examples of a trajectory per type of motions** Simulations were performed with N = 50 time points per trajectory, and time step t = 1/30. Parameters used for each motion class are: BM ($\sigma = 1$), OU ($\lambda = 0.5$, $\sigma = 1$), DIR ($\|u\| = 0.7$, $\sigma = 1$), FBM (FBM1 with $H = 0.2$, and FBM2 with $H = 0.8$), CTRW ($\sigma = 1$, $\gamma = 0.01$ for CTRW1 and $\gamma = 0.9$ for CTRW2).

OU The continuous-time OU process[9] suits particles with limited mobility due to an external force attracting them toward an equilibrium point[10]. It verifies

$$dX_t = -\lambda(X_t - \theta)dt + \sigma dB_t \tag{4}$$

where θ is the equilibrium point, λ the weight of the drift term, and σB_t defines the Brownian component of the motion. In the following, up to the change of variables $\tilde{X}_t = X_t - \theta$, we consider $\theta = (0, 0)$. Assuming that stochastic processes have independent components, the general results for dimension one apply.

The previous equation can be solved by variation of constants, obtaining

$$X_t = X_0 e^{-\lambda t} + \sigma \int_0^t e^{-\lambda(t-s)}\, dB_s \tag{5}$$

where X_0 is the initial position and the integral term on right side follows the normal distribution $\mathcal{N}(0, \frac{\sigma^2}{2\lambda}(1 - e^{-2\lambda t})\mathbf{I}_2)$. The covariance function[11] is:

$$\text{Cov}(X_t, X_s) \;=\; \left[\left(\text{Var}(X_0) - \frac{\sigma^2}{2\lambda}\right) e^{-\lambda(t+s)} + \frac{\sigma^2}{2\lambda} e^{-\lambda|t-s|}\right] \mathbf{I}_2 \tag{6}$$

showing that the process properties strongly depend on the statistical properties of X_0 (Var denotes the variance of a process).

Assuming that $X_0 \sim \mathcal{N}(0, \frac{\sigma^2}{2\lambda}\,\mathbf{I}_2)$ and that it is independent of B_t, we get that $X_t \sim \mathcal{N}(0, \frac{\sigma^2}{2\lambda}\mathbf{I}_2)$, proving that X_t is a continuous Gaussian stationary process. The increments are also normally distributed and we have that:

$$\text{Cov}(X_t - X_s, X_s) = -\frac{\sigma^2}{2\lambda}(1 - e^{-\lambda(t-s)})\mathbf{I}_2, \quad \forall t, s \in \mathbb{R}^+\,,\ t > s\,. \tag{7}$$

(7) proves that the increments are stationary but not independent because they are negatively correlated. We note that the variance of X_t is bounded, proving that the OU process exhibits a confined diffusion: if $\lambda << \sigma$, the motion will present a Brownian-like behavior, while, if $\lambda >> \sigma$, we obtain more confined trajectories.

Finally, by a straightforward computation and using the independence of X_0 and B_t, we obtain

$$\langle X_t^2 \rangle_{ens} \;=\; 2\text{Var}(X_0)(1 - e^{-\lambda t})^2 \;+\; \frac{\sigma^2}{\lambda}(1 - e^{-2\lambda t})\,,$$

$$\langle X_t^2 \rangle_{T,\Delta t} \;=\; \frac{2\sigma^2}{\lambda}(1 - e^{-\lambda \Delta t}) \;+\; 2\left(\text{Var}(X_0) - \frac{\sigma^2}{2\lambda}\right)(1 - e^{-\lambda \Delta t})^2 \frac{1 - e^{-2\lambda(T-\Delta t)}}{2\lambda(T - \Delta t)}\,.$$

confirming the OU process's subdiffusive and ergodic behavior[11,12] under the hypothesis $X_0 \sim \mathcal{N}(0, \frac{\sigma^2}{2\lambda}\,\mathbf{I}_2)$.

FBM It is a generalization of BM, starting at zero, and characterized by Gaussian increments that are stationary but not independent. This enables the modeling of particles moving in constrained or crowded environments[13].

Formally, FBM is a continuous Gaussian process with increments verifying[14–16]:

$$(X_t - X_s) \sim \mathcal{N}(0, |t - s|^{2H}\mathbf{I}_2)$$

where $H \in (0, 1)$ is the Hurst parameter which determines the intrinsic nature of this process. In particular, for $H = 1/2$ FBM reduces to BM.

For $H \neq 1/2$, as the variance of the increments depends only on the time interval and not on the current time, the process has stationary increments. However, as the variance of $X_t - X_0$ depends on t, it is not stationary. Moreover, for $t, s \in \mathbb{R}^+$ with $t > s$, it holds

$$\text{Cov}(X_t, X_s) = [t^{2H} + s^{2H} - |t-s|^{2H}], \quad \mathbb{E}[(X_t - X_s)X_s] = [t^{2H} - s^{2H} - |t-s|^{2H}]$$

showing that the increments are not independent and that the correlation of successive increments depends on H (positive for $H > 1/2$, negative for $H < 1/2$).

Finally, similarly to B_t, we obtain

$$\langle X_t^2 \rangle_{ens} = 2t^{2H}, \quad \langle X_t^2 \rangle_{T,\Delta t} = 2(\Delta t)^{2H}$$

which points out the ergodicity of the process and the variety of dynamics represented by FBM: superdiffusive for $H > 1/2$, and subdiffusive for $H < 1/2$.

CTRW This process has been introduced[17] to describe trapping for random walks, exhibiting trajectories alternating jumps and waiting times; after every jump, the trajectory maintains the same position for a duration equal to the related waiting time[18,19].

Denoting by $N(t)$ the number of jumps up to time t, by $\{t_i\}_{i=0}^{N(t)}$ the jump times, and assuming that $t_0 = 0$ and $X_{t_0} = (0,0)$, the position of a particle at time t is given by:

$$X_t = \sum_{i=1}^{N(t)} \xi_i$$

where ξ_i denotes the random jump and $\tau_i = t_i - t_{i-1}$ defines the waiting time between consecutive jumps. Jumps $\{\xi_i\}_i$ are independent and identically distributed (iid) random variables following a Gaussian distribution $\mathcal{N}(0, \sigma^2 \mathbf{I}_2)$, while the waiting times $\{\tau_i\}_i$ are iid random variables following a power law distribution with density $\psi(t) \sim t^{-\gamma-1}$ with $\gamma \in]0,1[$ (see[18]). Finally, we assume that the families $\{\xi_i\}_i$ and $\{\tau_i\}_i$ are independent.

We note that, for large γ, large waiting times are less likely to occur, and CTRW is then more similar to BM.

CTRW is a Gaussian process with independent increments, as X_t is the sum of Gaussian independent random variables. However, the variance of increments explicitly depends on the current time (see Chapter 4 in[18]), and it holds:

$$\mathbb{E}[(X_{t+\Delta t} - X_t)^2] = 2\sigma^2 \mathbb{E}[N(t+\Delta t) - N(t)] \propto [(t+\Delta t)^\gamma - t^\gamma] \tag{8}$$

proving that the increments are non-stationary and the process is not stationary either. Furthermore, because of jumps, a CTRW path is not continuous.

Finally, CTRW exhibits the following non-ergodic property[18,20–22]:

$$\langle X_t^2 \rangle_{ens} \propto t^\gamma, \quad \langle X_t^2 \rangle_{T,\Delta t} \propto \Delta t.$$

Ensemble and time averages point out different behaviors. The ensemble-average MSD is governed by the exponent of the power-law distribution for waiting times; as $\gamma \in]0,1[$, this indicates a subdiffusive trajectory. On the other side, the time-average MSD suggests a normal diffusion. According to the MSD criterion, a CTRW path is classified as BM, pointing out that MSD method is inadequate to describe CTRW trajectories.

**Process**	**Continuous**	**Gaussian**	**Stationary**	**Stationary Increments**	**Independent Increments**	**Ergodic**
**BM, DIR**	×	×		×	×	×
**OU**	×	×	×	×		×
**FBM**	×	×		×		×
**CTRW**		×			×	

Table S1: **List of main properties for each stochastic process.** Properties for OU process refer here to an initial state $X_0 \sim \mathcal{N}(0, \frac{\sigma^2}{2\lambda}\mathbf{I}_2)$; FBM refers to the case $H \neq 1/2$.

References

1. Einstein, A. (1905). On the motion of small particles suspended in liquids at rest required by the molecular-kinetic theory of heat. Annalen der Physik *17*, 549–560.

2. Qian, H., Sheetz, M.P., and Elson, E.L. (1991). Single particle tracking. Analysis of diffusion and flow in two-dimensional systems. Biophysical Journal *60*, 910–921.

3. Saxton, M.J., and Jacobson, K. (1997). Single-particle tracking: applications to membrane dynamics. Annual Review of Biophysics and Biomolecular Structure *26*, 373–399.

4. Briane, V., Vimond, M., and Kervrann, C. (2020). An overview of diffusion models for intracellular dynamics analysis. Briefings in Bioinformatics *21*, 1136–1150.

5. Weron, A., Janczura, J., Boryczka, E., Sungkaworn, T., and Calebiro, D. (2019). Statistical testing approach for fractional anomalous diffusion classification. Physical Review E *99*, 042149.

6. Birkhoff, G.D. (1931). Proof of the ergodic theorem. Proceedings of the National Academy of Sciences *17*, 656–660.

7. Krengel, U. (2011). Ergodic theorems vol. 6. Walter de Gruyter.

8. Ruan, G., Agrawal, A., Marcus, A.I., and Nie, S. (2007). Imaging and tracking of tat peptide-conjugated quantum dots in living cells: new insights into nanoparticle uptake, intracellular transport, and vesicle shedding. Journal of the American Chemical Society *129*, 14759–14766.

9. Uhlenbeck, G.E., and Ornstein, L.S. (1930). On the theory of the Brownian motion. Physical Review *36*, 823.

10. Monnier, N., Guo, S.M., Mori, M., He, J., Lénárt, P., and Bathe, M. (2012). Bayesian approach to MSD-based analysis of particle motion in live cells. Biophysical Journal *103*, 616–626.

11. Mardoukhi, Y., Chechkin, A., and Metzler, R. (2020). Spurious ergodicity breaking in normal and fractional Ornstein-Uhlenbeck process. New Journal of Physics *22*, 073012.

12. Cherstvy, A.G., Thapa, S., Mardoukhi, Y., Chechkin, A.V., and Metzler, R. (2018). Time averages and their statistical variation for the Ornstein-Uhlenbeck process: Role of initial particle distributions and relaxation to stationarity. Physical Review E *98*, 022134.

13. Guigas, G., Kalla, C., and Weiss, M. (2007). Probing the nanoscale viscoelasticity of intracellular fluids in living cells. Biophysical Journal *93*, 316–323.

14. Lawler, G.F. (2010). Stochastic calculus: An introduction with applications. American Mathematical Society.

15. Coutin, L. (2007). An introduction to (stochastic) calculus with respect to fractional Brownian motion. In Séminaire de Probabilités XL pp. 3–65.. Springer pp. 3–65.

16. Dieker, T. (2004). Simulation of fractional Brownian motion. Masters Thesis, Department of Mathematical Sciences, University of Twente.

17. Montroll, E.W., and Weiss, G.H. (1965). Random walks on lattices. II. Journal of Mathematical Physics *6*, 167–181.

18. Klafter, J., and Sokolov, I.M. (2011). First steps in random walks: from tools to applications. OUP Oxford.

19. Germano, G., Politi, M., Scalas, E., and Schilling, R.L. (2009). Stochastic calculus for uncoupled continuous-time random walks. Physical Review E *79*, 066102.

20. Lubelski, A., Sokolov, I.M., and Klafter, J. (2008). Nonergodicity mimics inhomogeneity in single particle tracking. Physical review letters *100*, 250602.

21. Jeon, J.H., and Metzler, R. (2010). Analysis of short subdiffusive time series: scatter of the time-averaged mean-squared displacement. Journal of Physics A: Mathematical and Theoretical *43*, 252001.

22. Neusius, T., Sokolov, I.M., and Smith, J.C. (2009). Subdiffusion in time-averaged, confined random walks. Physical Review E *80*, 011109.
